# Genotoxic and antigenotoxic medicinal plant extracts and their main phytochemicals: “A review”

**DOI:** 10.3389/fphar.2024.1448731

**Published:** 2024-11-29

**Authors:** Ghanya Al-Naqeb, Aliki Kalmpourtzidou, Francesca Giampieri, Rachele De Giuseppe, Hellas Cena

**Affiliations:** ^1^ Laboratory of Dietetics and Clinical Nutrition, Department of Public Health, Experimental and Forensic Medicine, University of Pavia, Pavia, Italy; ^2^ Department of Food Sciences and Nutrition, Faculty of Agriculture Food and Environment, University of Sana’a, Sana’a, Yemen; ^3^ Department of Clinical Sciences, Università Politecnica delle Marche, Ancona, Italy; ^4^ Research Group on Food, Nutritional Biochemistry and Health, Universidad Europea del Atlántico, Santander, Spain; ^5^ Clinical Nutrition and Dietetics Service, Unit of Internal Medicine and Endocrinology, ICS Maugeri IRCCS, Pavia, Italy

**Keywords:** plant extracts, genotoxic, non-genotoxic, antigenotoxic, phytochemicals

## Abstract

Many medicinal plant extracts have been proven to have significant health benefits. In contrast, research has shown that some medicinal plant extracts can be toxic, genotoxic, mutagenic, or carcinogenic. Therefore, evaluation of the genotoxicity effects of plant extracts that are used as traditional medicine is essential to ensure they are safe for use and in the search for new medication. This review summarizes 52 published studies on the genotoxicity of 28 plant extracts used in traditional medicine. A brief overview of the selected plant extracts, including, for example, their medicinal uses, pharmacological effects, and primary identified compounds, as well as plant parts used, the extraction method, genotoxic assay, and phytochemicals responsible for genotoxicity effect were provided. The genotoxicity effect of selected plant extracts in most of the reviewed articles was based on the experimental conditions. Among different reviewed studies, A total of 6 plant extracts showed no genotoxic effect, other 14 plant extracts showed either genotoxic or mutagenic effect and 14 plant extracts showed anti-genotoxic effect against different genotoxic induced agents. In addition, 4 plant extracts showed both genotoxic and non-genotoxic effects and 6 plant extracts showed both genotoxic and antigenotoxic effects. While some suggestions on the responsible compounds of the genotoxicity effects were proposed, the proposed responsible phytochemicals were not individually tested for the genotoxicity potential to confirm the findings. In addition, the mechanisms by which most plant extracts exert their genotoxicity effect remain unidentified. Therefore, more research on the genotoxicity of medicinal plant extracts and their genotoxicity mechanisms is required.

## 1 Introduction

Numerous extracts from plants and animals are essential for treating a variety of ailments (Keesing and Ostfeld, 2021). Many ancient and modern societies have relied heavily on medicinal plants for their medical needs. The World Health Organization estimates that between 70% and 80% of people worldwide mostly use traditional medicine, with 85% of that medicine derived from plants (Muhammad et al., 2011). In Western medicine, biodiversity plays a significant role with approximately 118 of the top 150 prescription drugs in the United States coming from plant sources, and over 1,300 medicinal plants are used in Europe, 90% of which are collected from wild resources (Balunas and Kinghorn, 2005). In addition, more than 25% of prescribed medications in developed countries are derived from wild plant species, and up to 80% of people in developing countries rely entirely on herbal medicine for their primary healthcare needs (Hamilton, 2004).

Despite extensive development of pharmaceutical synthesis methods, medicinal plants remain important sources of new active ingredients because plants can synthesize and produce constituents that are difficult to obtain via chemical synthesis (Bardoloi and Soren, 2022). Plant phytochemicals can also be used as prototypes for the synthesis of new drugs with similar biological and therapeutic activities, or they can be slightly modified to make them more effective or less toxic (Regner et al., 2011). The use of medicinal plants is rapidly expanding around the world because of the rising demand for natural health products, botanical drugs, and secondary metabolites of medicinal plants (Munari et al., 2010).

There is a general belief that plant medicines are safe based on their long-term application, which makes their use more prevalent and widespread (Sponchiado et al., 2016). Additionally, excessive use of natural plant extracts without adequate information on their toxicity may have negative health effects, indeed research has shown that many plants which are used either as food ingredients or in traditional medicine can be, toxic, and genotoxic (Ping et al., 2017), and mutagenic or carcinogenic (Suzanne et al., 2011).

Genotoxicity is defined as potentially harmful effects on genetic material caused by the induction of permanent transmissible changes in the amount or structure of nucleic acids (Ogura et al., 2008). Genotoxicity, broadly defined as “damage to the genome,” is a distinct and important type of toxicity because specific genotoxic events are considered cancer hallmarks (Ellinger-Ziegelbauer et al., 2009). The consequences of such genotoxicity damage to DNA could include disease development and/or predisposition, increased morbidity/mortality, changes in heritable characteristics, and impaired reproductive capacity (Lázaro et al., 2010). Genotoxicity test aims to yield information on all types of mutations, such as gene mutations, structural chromosome aberrations (clastogenicity), and numerical chromosome aberrations (aneugenicity). Different methodologies, strategies, and approaches have been developed to evaluate chemicals/phytochemicals that may have genotoxic effects. Standard tests battery has been developed which includes the assessment of mutagenicity in a bacterial reverse mutation test (Ames test) and genotoxicity in mammalian cells *in vitro* or *in vivo* using different methods including a comet, micronucleus (MN), and chromosomal aberration tests (CA) (Kirkland et al., 2005).

Genotoxicity tests are being undertaken in many laboratories across the world to raise awareness of potential risks, as most plant extracts used as traditional medicines have not been widely subjected to extensive toxicological tests like those necessary for modern pharmaceutical substances (Verschaeve, 2015). The presence of genotoxic chemicals in plant extracts raises concerns about the cancer risks associated with the plants’ long-term usage as medicines or food. Plant extracts that exhibit obvious genotoxic or mutagenesis capabilities should be regarded as potentially hazardous. Therefore, genotoxicity studies should make it possible to identify potentially hazardous plants, point out the need for additional research, and provide a warning that genotoxic plant extracts should at the very least be handled carefully (Verschaeve, 2015).

Genotoxicity tests can also reveal plant extracts that have anti-genotoxic and anti-mutagenic properties (Felício et al., 2011). Research has reported that some plant extracts, such as *Fraxinus angustifolia subsp.* syriaca (Boiss.) Yalt (Bouguellid et al., 2020), and *Equisetum arvense* L., (Dormousoglou et al., 2022), showed modulatory effects on mutagenesis and clastogenesis, the two genotoxic phenomena associated with carcinogenesis. Plants extract obvious anti-genotoxicity potential, on the other hand, may be assumed to be interesting for their chemo-preventive properties and potential therapeutic use. Anti-genotoxic agent is indeed a substance that reduces or counteracts the mutagenicity of physical and chemical mutagens (Bhattacharya, 2011). The study of antigenotoxic properties should be viewed in the context of the ongoing search for new anticancer drugs, which is necessary because most of the current anti-cancer drugs frequently have severe side effects, and replacement by at least as accurate but less hazardous substances is desired. Strong anti-genotoxic compounds may have the potential to be anticarcinogens because many carcinogens are also genotoxic. This clarifies the hunt for antigenotoxic qualities in plant extracts and the identification of the constituents that give them this attribute. These substances could then serve as the foundation for novel anti-cancer medications or functional food items (Verschaeve and Van Staden, 2008). Recent research has given credence to the identification of novel bioactive phytocompounds that counteract mutagenesis, as mutagens are involved in the initiation and promotion of cancer (Stoczynska et al., 2014).

Generally, research on plant extracts, especially toxicological research, is much less active compared to that of conventional drugs. This narrative review aims to summarize the most recent data on the genotoxicity and the non-genotoxicity properties of medicinal plant extracts and their main phytochemicals. A brief overview on these plant extracts including the medicinal uses and the main health benefits is discussed in this review. A summary of the plant parts used, the extraction method and solvent used for the extraction, the genotoxic assay used, the model organism/cell line used, and the main findings were also provided.

## 2 Materials and procedure

Selected articles from the 2010–2024 databases of ScienceDirect, PubMed, Web of Science, and Scopus were searched for relevant material for the current review using terms like “*in vitro*,” *in vivo*, “genotoxic, anti-genotoxicity,” or “non-genotoxic,” as well as “plants extracts” or “medicinal plants.” Most of the selected publications provided phytochemicals analysis of the plant extracts studied and suggested phytochemicals responsible for the genotoxicity effect.

## 3 Genotoxicity studies of plants extracts

The results of the literature search revealed a variety of articles that reported genotoxicity studies of plant extracts. A total of 28 plant extracts in 52 articles were included; these plants belong *to* 23 different families (Tables 1–3).

**TABLE 1:** Summary of genotoxic plants extract studies.

N	Author, Year, Country	Plant name	Family	Part used, extract	Concentration	Genotoxicity assay	Model organism/cell line	Positive control	Effect
1	De Quadros et al. (2023) Brazil/Portugal	*Rubus rosifolius* Sm.	Rosaceae	Methanolic extract of stem	0.01 and 100 μg/mL	*In vitro* CBMN and comet assay	HepG2/C3A	Dox, B [a]P	Genotoxic effect
2	Ramalho et al. (2023) Brazil	*Azadirachta indica* A. Juss.	Meliaceae	Ethanol/water extract of the leaves	300, 600 and 1,200 mg/kg	*In vivo* MN test	Bone marrow of Wistar rats	No PC was applied	Non-genotoxic effect
	Akaneme and Amaefule (2012), Nigeria	*Azadirachta indica* A. Juss.		Aqueous extract of leaves	10,15% and 20%	*In vivo* Allium cepa assay	The onion bulbs	No PC was applied	Mutagenic effect
3	de Barros et al. (2022) Brazil	*Moringa oleifera* Lam.	Moringaceae	Aqueous extract of leaves infusion and powder	2000 mg/kg	*In vivo* comet and MN tests	Whole blood cells from mice	MET 20 mg/kg	Non-genotoxic effect
	Bakare et al. (2022) Nigeria	*Moringa oleifera* Lam.		Aqueous extract of leaves	50–200 mg/kg	*In vivo* Allium cepa chromosome aberration	Onion bulbs and Somatic cells of Allium cepa and germ cells of male mice	CP 20 mg/kg	Genotoxic effect
	Asare et al. (2012) Ghana	*Moringa oleifera* Lam.		Aqueous extract of leaves	3,000 mg/kg	*In vivo* MN test	Bone marrow erythroblasts	ENU 20 mg/kg	Genotoxic effect
4	Ferreira et al. (2022) Brazil	*Eugenia uniflora* L.	Myrtaceae	Aqueous extract from leaves	1,000–2000 mg/kg	*In vivo* MN test	Murine erythrocytes of the mouse	Dox 40 mg/kg	No genotoxic effect
	da Cunha et al. (2016) Brazil	*Eugenia uniflora* L.		Ethanolic extract from leaves	1,000–2000 mg/kg	*In vivo* MN Test	Murine erythrocytes of the mouse	Dox 40 mg/kg	No genotoxic effect
	Kuhn et al. (2015) Brazil	*Eugenia uniflora* L.		Aqueous extracts from leaves, juices, fruit pulp and essential oil was obtained from fresh leaves.	6–24 mg/mL	*In vivo* Allium cepa test	Onion bulbs	GLY	Mutagenic activity of aqueous extract and the oil at low concentration, and at higher concentrations can be considered antimutagenic. No mutagenic activity for the juice extract
5	Quadros Gomes et al. (2021) Brazil	*Eleutherine bulbosa* (Mill.) Urb.	Iridaceae	Ethanol extract of bulbs and dichloromethane fraction and its active compound naphthoquinone	(2.45,4.90, 9.80) (2.38, 4.76, 9.52) (3.88, 7.77, 15.55) μg/mL	*In vitro* Comet Assay	HepG2 Cells	Dox 0.02 μg/mL)	Genotoxic effect
	Castro et al. (2021) Brazil	*Eleutherine bulbosa* (Mill.) Urb.		Isoeleutherin and eleutherin isolated	5.55, 7.77 and 3.88 μg/mL	*In vivo* Allium cepa and *in vitro* MN tests	Seeds of the species Allium cepa HepG2 cells	COL DOX 0.02 μg/mL	Genotoxic and mutagenic effects of eleutherin more than isoeleutherin at tested conditions
6	Tavares et al. (2011) Brazil	*Sapindus Saponaria* L.	Sapindaceae	Proteins of aqueous extract from seeds	3.7–300 μg/mL	*In vitro* Ames and CBMN tests	*Salmonella*/Microsome and HepG2	B [a]P 0.1 μg/mL	Mutagenic and genotoxic effects
7	Grujičić et al. (2020) Serbia	*Teucrium arduinii* L. and Teucrium flavum L.	Lamiaceae	Methanolic extract from stems, leaves, and flowers	125–1,000 μg/mL	*In vitro* CBMN and comet assays	Human lymphocytes cells	MMC 0.5 μg/mL	Genotoxic effect
8	Moreira Szokalo et al. (2020) Argentina	*Smallanthus sonchifolius* (Poepp.) H.Rob	Asteraceae	Aqueous extract of the aerial parts	CHO-K1 up to 66.7 μg/mL for 3 h, HepG2 cells up to 533.6 μg/mL for 24 h	*In vitro* CBMN assay	CHO–K1 and HepG2 cells	CP 4 mM	Genotoxic effect
9	Guo et al. (2021) China	*Fritillaria cirrhosa* D. Don	Liliaceae	Aqueous extract of the bulbus	20, 40, 80 and 160 μg/mL	*In vitro* CBMN assay	NCM460 cells	CYB 0.8 μg/mL	Genotoxic effect
	Guo et al. (2020) China	*Fritillaria cirrhosa* D. Don		Aqueous extract of the bulbus	20, 40, 80 and 160 μg/mL	*In vitro* CBMN Assay	NCM460 cells	CYB 0.8 μg/mL	Genotoxic effect
	Guo et al. (2017a) China	*Fritillaria cirrhosa* D. Don		Aqueous extract of the bulbus	20, 40, 80 and 160 μg/mL	*In vitro* CBMN assay	NCM460 cells	CYB 0.8 μg/mL	Genotoxic effect
10	Saravanan et al. (2020) India/United Kingdom	Kalanchoe pinnata (Lam.) Pers.	Crassulaceae	Ethanolic extract of Kalanchoe pinnata leaves	500 and 1,000 mg/kg	Ames test and *in vivo* MN	*Salmonella* Strain and *in vivo* mice bone marrow	CP 40 μg/mL	No mutagenic effect in Ames test, weak genotoxic *in vivo* MN assay in mice
	Bhavsar and Chandel, (2022) India	*Kalanchoe pinnata (Lam.) Pers.*		fresh *Kalanchoe pinnata* leaf juice	50 and 70 µL	*In vitro* CBMN assay	Human Peripheral blood	No PC	No genotoxic effect at 70 µL There was increase in MI only
11	Kahaliw et al. (2018) Ethiopia/Sweden	*Pterolobium stellatum (Forssk.) Brenan*	Fabaceae	Chloroform and methanol root extracts	0.01 mg/mL to 1 mg/mL.	*In vitro* comet assay	HepG2	CAT 3 mM	Genotoxic effect
12	Araújo et al., 2015 Brazil	*Euphorbia hyssopifolia* L.	Euphorbiaceae	Ethanol extract of aerial parts of stem, leaves	0.01, 0.1 and 1.0 mg/mL	*In vitro* comet assay and CBMN assay	Human HepG2 cells	MMS (4 × 10−4 M)	Genotoxic effect
13	Jacociunas, et al. (2013) Brazil	*Cynara scolymus* L.	Asteraceae	Aqueous extract of leaves	0.62, 1.25, 2.5 and 5.0 mg/mL	*In vitro* CBMN assay	Chinese hamster ovary cells (CHO)	EMS 0.75 μg/mL	Genotoxic effect
14	Santos et al. (2012) Brazil	*Bauhinia platypetala* Burch. ex Bent*h.*	Bauhinia	Leaves ethanolic extract and it’s an ethereal fraction	50 μg/mL	*In vitro* comet assay	V79 cells	MMS 40 µM	Genotoxic effect

CP = cyclophosphamide; DOX = doxorubicin, B [a]P =Benzo [a]pyrene, ENU = N-ethyl-N-nitrosourea, MMC = Mitomycin C, MMS, methyl methane sulfonate, H_2_O_2_ = hydrogen peroxide, CYB, Cytochalasin B, EMS = ethyl methane sulfonate; MET, methotrexate; COL = colchicine; GLY, glyphosate.

**TABLE 2:** Summary of antigenotoxic plant extracts studies.

N	Author, Year, Country	Plant name	Family	Part used, extract	Concentration	Assay	Model organism/cell line	Positive control	Effects and reported mechanisms
1	Nascimento et al. (2024) Brazil	*Croton blanchetianus* Baill.	Euphorbiaceae	Essential oil From the leaves	1,000 and 2000 mg/kg	*In vivo* MN test	Bone marrow cells from mice	CP	Anti-genotoxic effect by protection against DNA damage induced by CP ↓ the MN frequency to the level of the control animals
	de Oliveira et al. (2022a) Brazil	*Croton blanchetianus* Baill.		Ethyl acetate fractions from ethanol: water extract of the leaves	1,000 or 2000 mg/kg	*In vivo* MN and comet assays	Mice peripheral blood and bone marrow	DXR	No genotoxic effect
	Freitas et al. (2020) Brazil	*Croton blanchetianus* Baill.		Ethanolic extract of leaves	50, 100, and 200 mg/kg	*In vivo* MN and comet assays	Mice Bone marrow or peripheral blood	DXR	No genotoxic effect
2	Dormousoglou et al. (2022) Greece	*Equisetum arvense* L.	Equisetaceae	Aerial parts of using methanol, and ethanol/water	10, 50 and 100 μg mL of each extract	*In vitro* CBMN assay	Human lymphocytes cells	MMC	Anti-genotoxic effect, ↓MN formation significantly induced by the MMC
	Kour, et al. (2017) India	*Equisetum arvense* L.		Ethanolic extract of aerial part of the sterile stem	500 mg/kg	*In vivo* chromosome aberration and Mitotic Index assays	Bone marrow cells of mice	CP	No genotoxic effect, Antimutagenic effect
3	Mićović et al. (2021) Serbia	*Hyssopus officinalis* L.	Lamiaceae	Essential oil and methanol extract of flowering aerial parts	400 μg/mL	*In vitro* comet assay	Human peripheral blood leukocytes *in vitro*	H_2_O_2_	Anti-genotoxic effect by reducing significantly ↓ DNA damage caused by hydrogen peroxide pretreatment
4	Zor and Aslan, (2020) Turkey, Franco	*Nigella sativa* L.	Ranunculaceae	Seeds oil	1, 5, and 10 g/mL	*In vitro* MN assay	Human lymphocyte cell	CP	Anti-genotoxic effect measured by significantly ↓MN formation by protection the cells against cytogenetic damage caused by CP
	Nguyen et al. (2019) Morocco/Belgium	*Nigella sativa* L.		Aqueous seed extracts	0–18 mg/mL	*In vitro* comet assay and CBMN assay	Human C3A cells	MMS	No genotoxic was shown but some indications of both antigenotoxic and co-genotoxic effects were found
	Franco-Ramos et al. (2020) Mexico	*Nigella Sativa* L.		Seeds oil	500 mg/kg b.w	*In vivo* MN assay	Mice peripheral blood.	Cisplatin	Anti-genotoxic effect
5	Topalović et al. (2019) Serbia	*Oleaeuropaea* L.	Oleaceae	A standardized methanol dry olive leaf extract	0.125, 0.5 and 1 mg/mL	*In vitro* comet assay	Human whole blood	H_2_O_2_	Anti-genotoxic effect by reducing number of cells with estrogen-induced DNA damage by Synergistic antioxidant effect of the active compounds
	Topalović et al. (2015) Serbia	*Oleaeuropaea* L.		Standardized methanol dry olive leaf extract	0.125, 0.5 and 1 mg/mL	*In vitro* comet assay	Human peripheral blood leukocytes	H_2_O_2_	Anti-genotoxic by synergistic activation of several mechanisms such as ROS scavenging and stimulation of DNA repair effect
	Verschaeve et al. (2017) Belgium/Tunisia	Oleaeuropaea L.		Aqueous leaf extracts	5.0, 1.0, 0.2, mg/mL	*In vitro* comet assays	Human C3A hepatic cells	EMS	None of the extracts were found to be genotoxic
	Cabarkapa et al. (2011) Serbia	*Oleaeuropaea* L.		Commercial dry olive leaf extract	0.125, 0.5 and 1 mg/mL	*In vitro*, comet assay	Human leukocytes, induced with adrenaline and H_2_O_2_	H_2_O_2_	Anti-genotoxic By DNA protective effect against adrenaline and H_2_O_2_ by ROS Scavenging and enhancing cellular antioxidant capacity
6	Aprotosoaie et al. (2016) Romania	*Dracocephalum moldavica* L.	Lamiaceae	Hydro-methanolic extract from the aerial parts	25 and 100 μg/mL	*In vitro* comet and CBMN assays	Normal human dermal fibroblasts	Bleomycin	Anti-genotoxic effect by reduction of DNA damage ↓ MN The iron-chelating qualities, DNA repair processes, and free radical scavenging activity were suggested to be the mechanisms of the protective effects
7	Andrade et al. (2016) Brazil	*Solanum lycocarpum* A. St.-Hil.	Solanaceae	*Solanum lycocarpum* fruits glycoalkaloid extract	4, 8, 16 and 32 μg/mL.	*In vitro* comet and chromosomal aberrations assays	V79 cells	MMS	Anti-genotoxic effect by reduction of DNA damage and the chromosomal aberration frequency induced by MMS due to its major glycoalkaloids, solamargine and solasonine
	Tavares et al. (2011) Brazil	*Solanum lycocarpum* A. St.-Hil.		Hydroalcoholic extract *Solanum lycocarpum fruits*	16, 32, and 24 μg/mL, 0.25, 0.50, 1.0, and 2.0 g/kg	*In vitro* chromosomal aberrations and *in vivo* MN assays	V79 cells, bone marrow from Swiss mice	DOX	Not mutagenic effect, Antimutagenic effect
8	Diab et al. (2018) Egypt	*Salviaofficinalis* L.	Lamiaceae	Essential oil from leaves	0.1, 0.2, and 0.4 mL/kg)	*In vivo* comet assay and chromosomal aberrations test	Mouse bone marrow and male germ cells	CCl4	Non-genotoxic and anti-genotoxic effect by inhibition of the DNA-inducing agents before their attacking to DNA molecule, and the effect was proposed to be due to the presence of the monoterpenes’
	Nicolella et al. (2014) Brazil	*Salviaofficinalis* L isolated compound		Manool isolated from the methanol/water extracts and its hexane fraction of the leaves	0.5–8.0 μg/mL	*In vitro* CBMN Assay	V79 and HepG2 Cells	EMS	Genotoxic effect at the highest concentration in both V79 and HepG2 cells and antigenotoxic effect in HepG2 cells, at lower concentration by protective effect against chromosome damage induced by EMS
	Nicolella et al., 2021 Brazil	*Salviaofficinalis* L.		Manool compound	0.5 and 1.0 μg/mL 5.0 and 20 mg/kg	*In vitro* and *in vivo* MN assay	V79 cells mouse peripheral blood	DOX H_2_O_2_	Antigenotoxic effect at lower concentration by reduction of chromosomal damage
9	Dassprakash et al. (2012) India	*Punica granatum* L.	Lythraceae	Aqueous leaves extract	400, 600 and 800 mg/kg body weight	*In vivo* MN test	Mouse bone marrow from Swiss albino mice	CP	Anti-genotoxic effect by reduction of micronucleated cells by antioxidants effects
10	Guo et al. (2017b) China	*Phyllanthus emblica* L.	Euphorbiaceae	Aqueous leaves extract of dried fruits	80, 160, and 320 μg/mL	*In vitro* CBMN assay	NCM460 normal colonic cells	MMC	Anti-genotoxic effect by reduction of DNA damage biomarkers Including MN, nucleoplasmic bridges, and nuclear buds in and genome protection from instability
	Guo and Wang (2016) China	*Phyllanthus emblica* L.		Aqueous leaves extract of dried fruits	160 μg/mL		Human NCM460 normal colon epithelial cells	DDP	Antigenotoxic effects by preventing mitosis exit through activation of the spindle assembly checkpoint in human NCM460 normal colon epithelial cells
	Guo et al. (2013) China	*Phyllanthus emblica* L.		ethanolic extract of dried fruits	0, 20, 40, 80, or 160 μg/mL	*In vivo* Micronucleus test	Human Colon Cancer Cells COLO320	Nocodazole	Genotoxic effects in cancer cells by enhancing the chromosomal instability biomarkers including the frequencies of MN, nucleoplasmic bridges with geraniin as the proposed principal compound
	Guo et al. (2018) China	*Phyllanthus emblica L.* active compound *geraniin*		Active compound geraniin	0–100 μg/mL	*In vitro* CBMN assay	NCM460 and COLO320 cells	7,12-dimethylbenz(a)anthracene (DMBA)	Genotoxic effects in cancer cells with chromosomal instability and antigenotoxic effects in norma cell

**TABLE 3:** Summary of genotoxic/antigenotoxic plants extract studies.

N	Author, Year, Country	Plant name	Family	Part used, extract	Concentration	Genotoxicity assay	Model organism/cell line	Positive control	Effects and reported mechanisms
1	Jakovljević et al. (2020) Serbia	*Artemisia vulgaris* L. and *Artemisia alba* Turra.	Asteraceae	Methanol extracts of aerial flowering parts	50, 100 and 250 μg/mL	*In vitro* CBMN assay	PBLs cells	MMC 0.5 μg/mL	Genotoxic and antigenotoxic effects, the anti-genotoxic effect of
2	Slapšytė et al. (2019) Lithuania	*Gratiola officinalis* L.	Plantaginaceae	Methanol and acetone extracts from the whole plant	100–500, in Ames test 50–250 μg/mL in Cometa ssay 12.5–50 μg/mL	*In vitro* Ames CBMN and comet assays	*S. typhimurium* Human lymphocytes cells	Dox 0.02 μg/mL, H2O2, 20 μM	Mutagenic effect at 100 μg/plate in Ames test. Genotoxic effect at 37.5 μg/mL
	Durnova and Kurchatova, (2015) Russia	*Gratiola officinalis* L.		Flavonoids extract from *Gratiola officinalis*	200 mg/kg	*In vitro* MN assay	Peripheral blood erythrocytes from mice	CP	Antimutagenic properties at 200 mg/mL
3	Moretti et al. (2013) Italy	*Dendrobium speciosum* Sm.	Orchidaceae	Leaves and stems methanol extracts o	2.5–10, μg/mL for steme,25–100 μg/mL for the leave extract	*In vitro* comet assay	HepG2 cells	4-NQO 3 μM	Anti-genotoxic effect and slightly genotoxic at the higher concentration
4	Ananthi et al. (2010) India	*Hemidesmus indicus (L.) R.Br.*	Asclepiadaceae	Ethanolic extract of *Hemidesmus indicus* roots	2–32 μg/mL	*In vitro* chromosome aberrations test	Human peripheral lymphocytes induced by cisplatin	Cisplatin	Anti-genotoxic effect at lower concentrations probably genotoxic at higher doses

### 3.1 *Croton blanchetianus* Baill. [Euphorbiaceae]

*Croton blanchetianus* Baill., is a member of the Euphorbiaceae family found in Brazil. The stem bark and leaves are often used to treat gastrointestinal diseases, inflammation, urethral discomfort (de Oliveira A. M. et al., 2022), and rheumatism. *Croton blanchetianus* Baill essential oil was found to have acaricidal activity against rhipicephalus (Rodrigues et al., 2019). In a recent study reported by Nascimento et al. (2024), an *in vivo* MN test was conducted to investigate the antigenotoxicity activity of the essential oil of *Croton blanchetianus* Baill., leaves on bone marrow cells from Swiss mice induced with cyclophosphamide (CP), a well-known genotoxic agent.

Treatment with essential oil of *Croton blanchetianus* Baill., leaves did not substantially increase the number of micronucleated polychromatic erythrocytes in the mice bone marrow cell when compared to the control CP indicating that, the essential oil was safe and non-genotoxic (Nascimento et al., 2024). In addition, the essential oil protected mouse blood cell DNA from CP action and significantly reduced micronucleated polychromatic erythrocytes. The chemical composition of the essential oil revealed that the terpene class was prominent, with the majority occurrence of mono and sesquiterpenes. The major phytochemicals found were α-pinene, β-phelandrene, terpinolene and germacrene. The anti-genotoxic action of the essential oil of the *Croton blanchetianus* Baill., was proposed to be due to the presence of these phytochemicals (Nascimento et al., 2024). de Oliveira A. M. et al. (2022), has reported that the ethyl acetate fractions obtained from ethanol water extract of *Croton blanchetianus* Baill., leaves did not show any genotoxic effect *in vivo* in male Swiss mice peripheral blood and bone marrow cells when assayed using MN and comet assays.

Furthermore, the genotoxicity of an ethanolic extract of *Croton blanchetianus* Baill., leaves was assessed in mouse bone marrow or peripheral blood cells using MN and comet tests (Freitas et al., 2020). The results showed that the extract was safe and non-genotoxic under the testing conditions. When compared to doxorubicin (DXR) agent as a positive control, *Croton blanchetianus* leaf extract did not cause MN or DNA damage at all of the doses or time frames studied. The phytochemicals examination of *Croton blanchetianus* Baill., leaves revealed the presence of alkaloids, terpenes/steroids, cinnamic acids, condensed tannins, flavonoids, saponins, and reducing sugars (Freitas et al., 2020).

### 3.2 *Rubus rosifolius* Sm. [Rosaceae]

*Rubus rosifolius* Sm., belongs to the Rosaceae family (Kanegusuku et al., 2007). Several studies have highlighted the traditional medicinal uses and therapeutic effects of *Rubus rosifolius* Sm., aerial parts including antibacterial, diuretic and antinociceptive properties (Petreanu et al., 2015), with an emphasis on the gastroprotective effect of the stem extract when compared to omeprazole (de Souza et al., 2017). Methanol extract from *Rubus rosifolius* Sm., steam showed genotoxic effect on human hepatoma cells (HepG2/C3A) when assayed using *in vitro* cytokinesis blocked (CBMN) and comet assays (De Quadros et al., 2023). Considering the experimental conditions, the steam extract caused significant DNA damage at a concentration of 1 μg/ml as detected by comet assay and caused an increase of cells with MN, nucleoplasmic bridges and nuclear buds at a concentration of 10 μg/ml as detected by CBMN assay.

The chemical characterization of the methanolic steam extract of from *Rubus rosifolius* Sm., evealed the presence of saponins and steroidal, including niga-ichigoside, quercetin glucuronide, tormentic acid, and 5,7-dihydroxy-6,8,4′-trimethoxyflavonol were found to be the major constituents in this extract (De Quadros et al., 2023). As authors indicated, one or more of these substances might be responsible for the genotoxic effect that was observed (De Quadros et al., 2023). Other plant species in the *Rubus* genus Sm., showed genotoxic effects. Alves et al. (2014) reported a genotoxic effect of *Rubus imperialis* Sm., extract on mouse bone marrow cells. Tolentino et al. (2015) reported a genotoxic effect on mice bone marrow cells treated with an extract of *Rubus niveus* Sm., aerial parts at a high concentration of 2000 mg/kg.

### 3.3 *Azadirachta indica* A. Juss. [Meliaceae]

*Azadirachta indica* A. Juss., which is known as neem, is one of the medicinal plants that is well documented as herbal medications. It belongs to the Meliaceae family and is native to India and Myanmar, as well as Bangladesh, Sri Lanka, and other nations (Ramalho et al., 2023). Various parts of *Azadirachta indica* A. Juss., including the bark, root, flowers, fruits, leaves, and seeds, are utilized in traditional medicine. The effects of plant leaves have been documented in the literature for a variety of purposes, including antitumor, hypoglycemic, anti-inflammatory, and antimicrobial activities (Santos et al., 2018). Ethanol extract of *Azadirachta indica* A. Juss., leaves at dosages of 300, 600, or 1,200 mg/kg did not cause any genotoxic effects in Wistar rats bone marrow as assayed by MN test (Ramalho et al., 2023). Rutin and quercetin were identified in leaf extract being rutin was the major flavonoid (Ramalho et al., 2023). On the other hand, another study reported by Akaneme and Amaefule (2012) using the *in vivo* Allium cepa assay showed that the aqueous leaf extracts of *Azadirachta indica* A. Juss., had a mutagenic effect. The different effect of the Azadirachta *indica* A. Juss., leaves extract in the 2 studies might be due to the different assay used, different extract applied, and different concentrations used.

### 3.4 *Moringa oleifera* Lam. [Moringaceae]

*Moringa oleifera* Lam., a member of the Moringaceae family, is a well-known medicinal plant found throughout Asia, Africa, and South America (Ayerza, 2011). *Moringa oleifera* Lam leaves have a high nutritional content and are considered a sustainable alternative food supplement for malnourished people, particularly in developing countries (Gupta et al., 2017). Several pharmacological activities of the leaves were reported including anticancer (Khor et al., 2018), anti-inflammatory, neuroprotective and radioprotective properties (Sulaiman et al., 2008).

*Moringa oleifera* Lam leaf infusion and powder did not cause genotoxic or mutagenic damage in mice when used at 2000 mg/kg as assayed using both comet and MN assays (de Barros et al., 2022). In another study, aqueous extract of *Moringa oleifera* Lam was genotoxic when tested using Allium cepa and male mouse germ cells (Bakare et al. (2022). When 0.5%–20% of the *Moringa oleifera* extract was applied to onion bulb roots, it significantly inhibited the growth of the roots, decreased the mitotic index (MI), and increased the Allium cepa chromosome aberration in comparison to the negative control bulbs. In addition, aqueous extract of *Moringa oleifera* Lam produced DNA damage in the sperm head and interfered with spermatogenesis of Swiss male mice given the aqueous extract (50–800 mg/kg) orally for 35 days. This shows that *Moringa oleifera* Lam may have the ability to affect male fertility and modify the somatic cell cycle (Bakare et al., 2022). Furthermore, oral administration of 3,000 mg/kg of aqueous *Moringa oleifera* Lam leaf extract, increased the number of micronucleated polychromatic erythrocytes in rats (Asare et al., 2012). The different studies on *Moringa oleifera* Lam leaves showed different genotoxic effects, that might be due to the different preparation of the extract, type of the assay used and the concentrations applied. Polyphenols are the major phytochemicals in the *Moringa oleifera* Lam leaves powder and infusion including cinnamic derivatives, caffeic acid, chlorogenic acid and rutin (de Barros et al., 2022). Phytochemicals of Moringa oleifera leaves such as tannins, phenolic acid, glucosinolates, isothiocyanates, saponins, flavonoids, steroids, terpenes, and alkaloids were also reported (Vergara-Jimenez et al., 2017). More research is needed to identify the genotoxic responsible compounds of *Moringa oleifera* Lam leaf extracts.

### 3.5 *Eugenia uniflora* L. [Myrtaceae]

*Eugenia uniflora* L., belongs to the Myrtaceae family, a native species of Brazil (Toscano et al., 2016). In folk medicine, the leaves of this plant are used to cure diarrhea, inflammatory diseases, hyperglycemia, and hypertension (Ferreira et al., 2022). Scientific studies have also demonstrated the antimicrobial (da Cunha et al., 2016), antinociceptive, anti-inflammatory and antioxidant activities (de Melo Candeia et al., 2022). Phytochemical analysis of the leaves of *Eugenia un*iflora L., revealed a high concentration of phenolic compounds including gallic acid, ellagic acid, and the flavonoid myricitrin (Ferreira et al., 2022). These polyphenolic compounds are commonly linked to the biological properties of the plant (Falcão et al., 2018). The aqueous extract of *Eugenia uniflora* did not induce MN in mice erythrocytes at the concentrations tested, exhibiting a profile like the vehicle-treated group after 24 and 48 h of treatment suggesting that there was no genotoxic activity *in vivo* under the experimental conditions (Ferreira et al., 2022). In addition, another study using ethanol extract of *Eugenia uniflora* L., leaves had no damage effects on DNA of human peripheral leukocytes (da Cunha et al., 2016). Furthermore, Kuhn et al. (2015) studied the mutagenic and antimutagenic effects of aqueous extracts from leaves, juices, fruit pulp and essential oil from fresh leaves through the Allium cepa test. Findings indicated that the aqueous extract and the oil of *Eugenia uniflora* L., have mutagenic activity at low concentrations used (6 mg/mL) and extracts at higher concentrations (24 mg/mL) can be considered antimutagenic. The juice extracts with and without seeds do not have mutagenic activity. As from the above studies, the different effects of *Eugenia uniflora* L., extracts were based on the extraction type, the assay used, and the concentrations applied.

### 3.6 *Equisetum arvense* L. [Equisetaceae]

*Equisetum arvense* L., a member of the Equisetaceae family, is known for its widespread use in traditional medicine (Garcia et al., 2012). Flavonoids, phenolic acids, alkaloids, phytosterols, tannins, and triterpenoids are among the physiologically active phytochemicals found in this plant (Cetojević-Simin et al., 2010). Dormousoglou et al. (2022), investigated the antigenotoxic effects of Soxhlet’s prepared extracts from aerial parts of the *Equisetum arvense* L., using different solvents including methanol, ethanol, water, and ethanol/water. The antigenotoxic potential was assayed by CBMN on human lymphocytes. Different extracts from the aerial parts of the *Equisetum arvense* L., were able to significantly reduce the production of MN in human cells induced with the well-known genotoxic agent mitomycin (MMC) (Dormousoglou et al., 2022). Methanol/water extract had the highest antigenotoxic activity, which was found to be rich in flavonoids, flavonoid-O-glycosides, phytosterols, phenolic and fatty acids, minerals, and primarily in K, Ca, Mg, Si, and P. The synergetic effect of these phytochemicals was proposed to be responsible for the anti-genotoxic effect (Dormousoglou et al., 2022). In addition, Kour et al. (2017) examined the antigenotoxic effect of ethanolic extract of the aerial part of the sterile stem of *Equisetum arvense* L., in mouse bone marrow cells stimulated with CP. The ethanol extract of *Equisetum arvense* was found to not affect the induction of chromosomal abnormalities and elevated mitotic index. *Equisetum arvense* L., ethanol extract exhibited antimutagenic and anticlastogenic properties in response to CP mutagenic effects in mice. The main components found in the ethanolic extract of the aerial section of the stem of *Equisetum arvense* L., were 2,6,10-trimethyl, 14-ethylene-14-pentadecne, n-hexadecanoic acid, phytol, and oleic acid. The antimutagenic and anticlastogenic effect of this extract was proposed to be due to affecting metabolic pathways, being antioxidant or acting on DNA replication (Kour et al., 2017).

### 3.7 *Hyssopus officinalis* L. [Lamiaceae]

**FIGURE 1:**
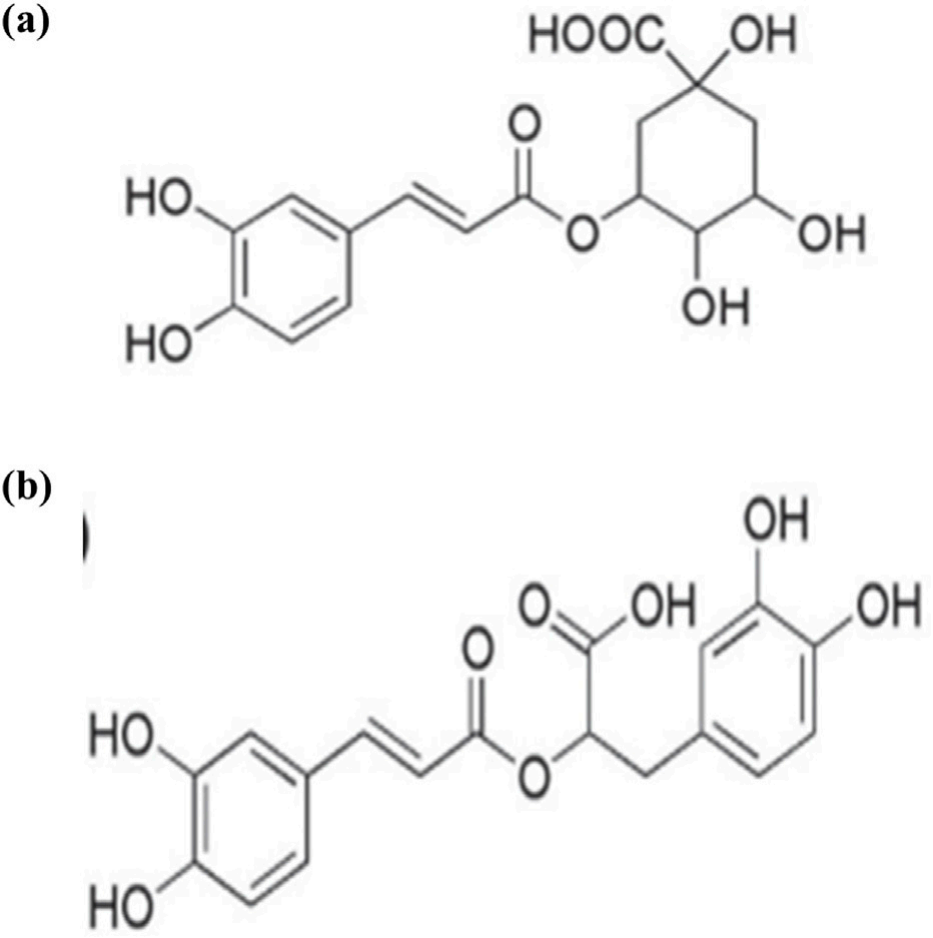
structure of **(A)** chlorogenic acid, **(B)** rosmarinic acid (Chaowuttikul et al., 2020).

*Hyssopus officinalis* L., belonging to the Lamiaceae family, is a herbaceous plant found primarily in the Mediterranean region (Džamic et al., 2013). It has been used as traditional medicine since ancient times and utilized in the food and cosmetics industries due to its aromatic properties (Venditti et al., 2015). The most important and widely studied product of *Hyssopus officinalis* L., is essential oil; extensive experimental evidence reported the antioxidant and anti-inflammatory antiulcer (Paun et al., 2014), and antidiabetic activities (Miyazaki et al., 2003). Mićović et al. (2021) used the comet assay to examine the antigenotoxic effect of *Hyssopus officinalis* L., essential oils and methanolic extract on human whole blood cells induced with hydrogen peroxide (H_2_O_2_). At 400 g/mL, essential oils and methanolic extracts of *Hyssopus officinalis* L. significantly decreased DNA damage induced by H_2_O_2_ pretreatment. The essential oil was found to be rich in monoterpene hydrocarbons including limonene, oxygenated monoterpenes and phenylpropanoids (Mićović et al., 2021). Analysis of methanol extracts flowering aerial parts revealed the presence of phenolic phytochemicals, specifically benzoic acid derivative (syringic acid), hydroxycinnamic acid derivatives including chlorogenic, feruloylquinic and rosmarinic acids, as well as caffeoyl pentoside and flavonoids derivatives of quercetin and diosmetin (Mićović et al. (2021). The considerable antigenotoxic action of the essential oil and methanolic extract could be relevant to the extract hydroxycinnamic acid derivatives. Previous studies have shown that chlorogenic and rosmarinic acids (Figure 1) were effective in reducing DNA damage (De Oliveira et al., 2012).

### 3.8 *Eleutherine bulbosa* (Mill.) Urb. [Iridaceae]

**FIGURE 2:**
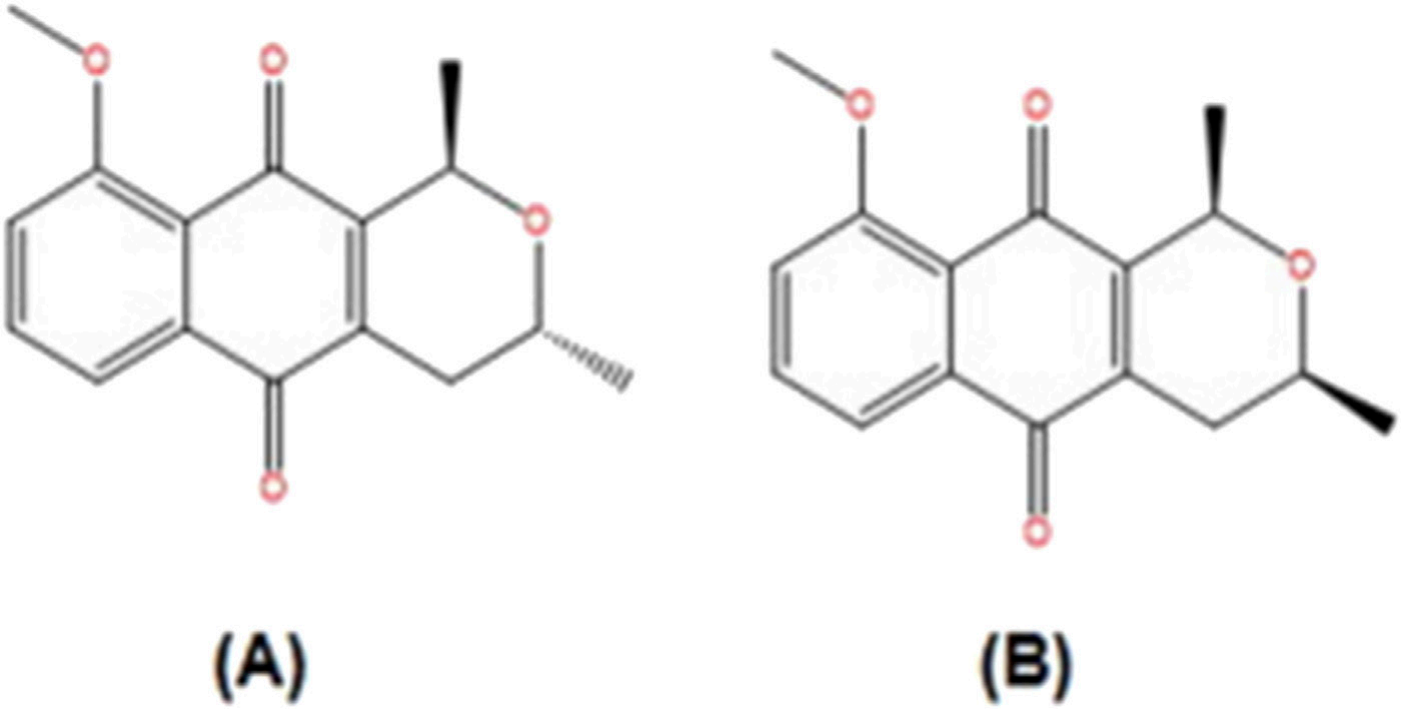
Structure of constituents from Eleutherine plicata.: **(A)** isoeleutherin; **(B)** eleutherin (Adapted from Castro et al. (2021)).

Eleutherine bulbosa (Mill.) Urb., belongs to the Iridaceae family and is widely distributed in Brazil. It has been demonstrated to be a promising medicinal plant, having activity linked to its quinones naphthoquinones compounds (Quadros Gomes et al., 2021). This plant is widely used to treat malaria, gastrointestinal illnesses, and diseases caused by bacteria, fungi, and other parasites (Couto et al., 2016), anemia, and blood purification (Odonne et al., 2013). Among the quinones identified in this plant are eleutherinone, isoeleutherol naphthoquinones eleutherin and isoeleutherin (Malheiros et al., 2015). Quadros Gomes et al. (2021) reported that methanolic extract and its dichloromethane fraction from the bulbs of *Eleutherine plicata* (Mill.) Urb., with its main phytochemicals isoeleutherin caused DNA damage in HepG2 cells as evaluated by comet assay. It was hypothesized that *Eleutherine plicata* (Mill.) Urb., genotoxicity is caused by naphthoquinone chemicals such as eleutherin and isoeleutherin (Figure 2) and involves many signaling pathways, such as oxidative stress, induction of apoptosis, and DNA alkylation (Quadros Gomes et al., 2021). In addition, Castro, et al. (2021) reported the evaluation of the genotoxicity and mutagenicity of isoeleutherin and eleutherin isolated from this plant using Allium cepa and MN tests. In the Allium cepa model, eleutherin caused a higher percentage of chromosomal aberrations than isoeleutherin, when compared to the positive control, eleutherin caused a higher rate of chromosomal aberrations. In addition, isoeleutherin showed MN frequency with direct relationship to concentration used. Oxidative stress might be linked to genotoxicity effect of naphthoquinone, isoeleutherin (Castro, et al., 2021), as these phytochemicals were reported to induce reactive oxygen species (ROS) and produced deplete glutathione, in human breast cancer cells (MCF-7) (Lin et al., 2007).

### 3.9 *Sapindus saponaria* L. [Sapindaceae]

*Sapindus saponaria* L., is a traditional medicine plant reported for the treatment of inflammations and injuries, diuretics and digestive tonic, antitumor effects (Rashed et al., 2013). *Sapindus saponaria* L., seed extract has been used as an antibacterial and antioxidant agent (Niloufer and Lakshmi, 2021). Tavares et al. (2011) reported that proteins of aqueous extract from *Sapindus saponaria* L., seeds exhibited mutagenicity as assayed by Ames test in the presence of metabolic activation. In addition, the genotoxic effect of the extract was detected in HepG2 cells using CBMN. The extract significantly increased the MN frequencies at all tested concentrations (3–300 μg/mL) and decreased the nuclear division indexes (NDI) in a dose dependent manner by nuclear breakages and cell cycle arrest. The presence of hydrolase enzymes including serine protease and a cysteine protease inhibitor from protein extract of *Sapindus Saponaria* L*.,* might be responsible for the genotoxic effects (Bocayuva Tavares et al., 2021).

### 3.10 *Artemisia vulgaris* L*.* and *Artemisia alba* Turra [Asteraceae]

*Artemisia vulgaris* L. and *Artemisia alba* Turra belong to the family Asteraceae, which are widely used in Europe, Asia, and North America, and are known as a source of traditional remedies (Oberprieler, 2001). Numerous biological actions for the genus *Artemisia* have been reported, including anti-malarial (Kane et al., 2019), anticancer (Gordanian et al., 2014), and anti-inflammatory and antioxidant activities (Abdul et al., 2018). The biologically active phytochemicals including flavonoids, coumarins, monoterpenes, lactones, sterols, and sesquiterpene lactones, that exhibit a wide range of biological effects and contribute to their practical application, were highlighted by previous pharmacological investigations of *Artemisia* species (Trendafilova et al., 2018). Methanol extracts of aerial flowering parts of *Artemisia vulgaris* L. and *Artemisia alba* Turra were tested for their genotoxicity in human peripheral blood lymphocytes (PBLs) using a CBMN assay (Jakovljević, et al., 2020). Both extracts significantly enhanced MN frequency in PBLs cells and significantly influenced the NDI compared to the negative control. Both extracts also increased the total number of cells with DNA damage as detected by comet assay. The presence of phenolic compounds including quercetin-3-O-glucopyranoside as the most abundant polyphenolic compounds in both extracts, could be a significant factor in their genotoxic action (Jakovljević et al., 2020). Engen et al. (2015), reported that quercetin and its natural glycosides increased sister chromatid exchanges and MN in Chinese hamster ovarian cells (V79). On the other hand, both extracts of *Artemisia vulgaris* L. and *Artemisia alba* Turra showed an anti-genotoxic effect when used as a treatment against mitomycin-C (MMC), the extracts decreased MN frequencies and NDI values significantly in compression to the MMC positive control in a dose-dependent manner. The anti-genotoxic effect of both extracts might be due to the higher chlorogenic acid content in both extracts which is considered as a significant factor in the protective activity against MMC-induced genotoxicity observed (Jakovljević et al., 2020).

### 3.11 *Teucrium arduini* L. and *Teucrium flavum* L. [Lamiaceae]

**FIGURE 3:**
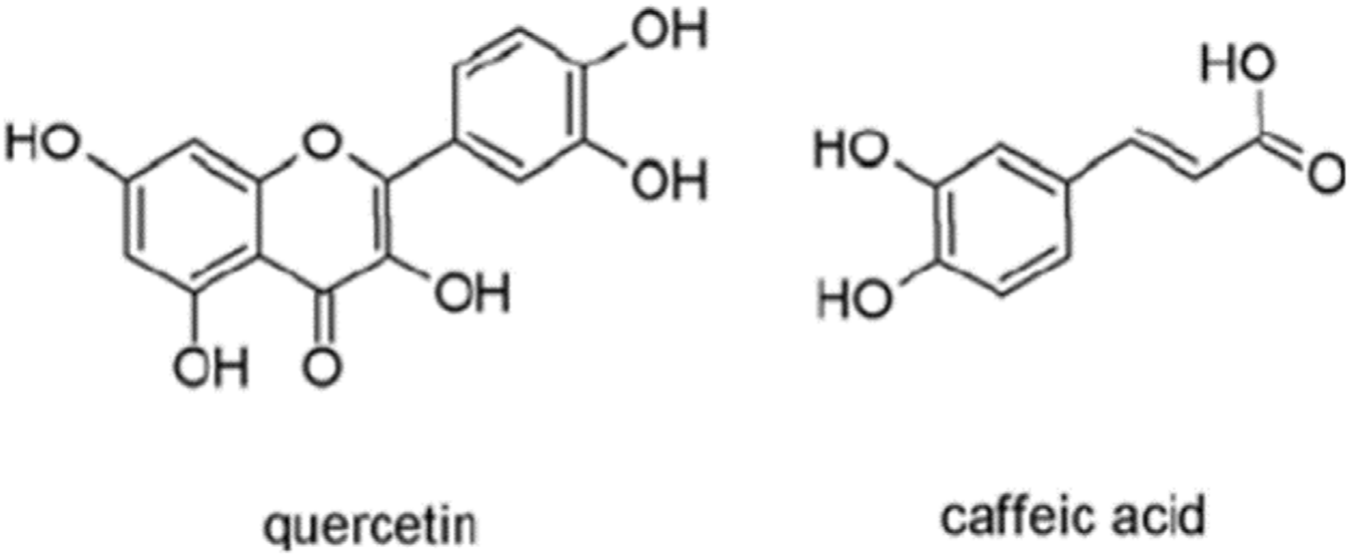
Structure of quercetin and caffeic acid (adapted from Kurt et al., 2023).

*Teucrium* spices belong to the Lamiaceae family and are widely found in the Mediterranean area (Grujičić et al., 2020). Many species belonging to the genus *Teucrium* are used in traditional medicine for the treatment of different diseases (Bellomaria et al., 1998). Among these spices, *Teucrium arduini* L., and *Teucrium flavum* L., are the most studied spices (Grujičić et al., 2020). *Teucrium arduini* L., has been reported to show antimicrobial (Kremer et al., 2013). *Teucrium flavum* L., was also found to have anti-inflammatory and antioxidant effects (Dall’Acqua et al., 2008). The genotoxic effect of methanolic extract from stems, leaves, flowers of *Teucrium arduini* L., and *Teucrium flavum* L., was determined in human lymphocyte cells using CBMN and comet assay (Grujičić et al., 2020). Both plants extract showed genotoxic over a dose range of 250–1,000 μg/mL. Methanol extracts from both plants showed a dose-related increase in MN frequencies and significantly increased the genetic damage index values, in comparison to the negative control as detected by comet assay. The polyphenolic and flavonoids composition of both extracts was qualitatively similar and caffeic acid and quercetin were the most commonly present phytochemicals (Grujičić et al., 2020). The observed genotoxic effect was suggested to be attributed to the individual or synergistic effects of complex mixtures of phenolic and flavonoid phytochemicals including caffeic acid and quercetin (Figure 3). According to these findings, both plant species phytochemicals may be genotoxic and further research is necessary before these plant extracts can be used safely on humans.

### 3.12 *Smallanthus sonchifolius* (Poepp.) H.Rob. [Asteraceae]

*Smallanthus sonchifolius* (Poepp.) H. Rob. known as Yacon, belongs to the Asteraceae family. It is a crop that is used in traditional folk medicine to treat diabetes, digestive and renal disorders (Sugahara et al., 2015). *Smallanthus sonchifolius* (Poepp.) H. Rob. pharmacological qualities have drawn more attention in recent years, primarily due to its potential as a hypoglycemic drug. As a result, the cultivation of this species has been expanded to several countries such as Italy, France, Germany, United States, Czech Republic, Russia, and Japan (Schorr et al., 2007). Yacon leaves, which are dried and used to make tea, have been shown to have anti-diabetic properties (Genta et al., 2010). Previous biological research has shown anti-inflammatory, antifungal, and antibacterial properties (Lin et al., 2003). In Yacon leaves, various phytochemicals such as phenolic acid, related diterpenoid substances, acetophenone phytoalexins, and sesquiterpene lactones have been identified (Frank et al., 2013). Literature data indicated that oral use of Yacon leaves was not recommended due to renal toxicity attributed to a class of terpenes called sesquiterpene lactones present in the aqueous extract (Barcellona et al., 2012).

In CHO-K1 cells, increasing concentrations of the aqueous extract from Yacon aerial parts up to 66.7 μg/mL for 3 h induced a significant increase in the frequency of MN, nuclear buds, and nucleoplasmatic bridges, whereas in HepG2 cells, a statistically significant increase in the frequency of MN was observed with tested concentrations of 133.4, 266.8, 400.2, and 533.6 μg/mL for 24 h indicating the genotoxic effect of the aqueous extract of the aerial parts of *Smallanthus sonchifolius (Poepp.) H. Rob.* (Moreira Szokalo et al., 2020). The chemical analysis of the aqueous extract of *Smallanthus sonchifolius* (Poepp.) H. Rob. revealed the presence of terpenoid phytochemicals including sesquiterpene lactones enhydrin and the dimer enhydrofolin, as the main metabolites together with phenolic metabolites (Moreira Szokalo et al., 2020). The *in vitro* genotoxic effect of *Smallanthus sonchifolius* (Poepp.) H. Rob. extract was suggested to be induced by either a direct or an indirect effect of phytochemicals present in the extract under the experimental conditions (Moreira Szokalo et al., 2020).

### 3.13 *Fritillaria cirrhosa* D. Don [Liliaceae]

*Fritillaria cirrhosa* D. Don*,* which belongs to the Liliaceae family, is a Chinese native plant which has been used extensively as a food and traditional medicine (Hao et al., 2013). Scientific research on *Fritillaria cirrhosa* D. Don has primarily focused on antitussive, expectorant, and antiasthmatic activities, anti-inflammatory and analgesic properties (Xu et al., 2016). These effects have been attributed to the plant’s principal phytochemicals, alkaloids including imperialine, peimisine, chuanbeinone, verticinone and verticine as the major phytochemicals of the plant (Wang et al., 2014; Xu et al., 2016). Almost all the alkaloids found in the bulbus *Fritillaria cirrhosa* D. Don aqueous extract are steroidal alkaloids (Hao et al., 2013). Steroidal alkaloids are a type of secondary metabolite that showed anticancer, antimicrobial, anti-inflammatory, and antinociceptive properties (Jiang et al., 2016). Aqueous extract of the bulbus from *Fritillaria cirrhosa* D. Don caused cytokinesis and mitotic abnormalities and increased in MN in normal human colon epithelial cell line (NCM460) (Guo X. H. et al., 2017). In addition, the same aqueous extract induced cytokinesis failure in NCM460 cells (Guo et al., 2020) and induced multipolar spindles mainly by promoting centrosome fragmentation (Guo et al., 2021). The genotoxic effect of the aqueous extract of bulbus *Fritillaria cirrhosa* D. Don was suggested to be related to its alkaloids, terpenoids and glycosides phytochemicals (Guo et al., 2020). Genotoxic effect of alkaloid rich extract from other plants was reported (Traore et al., 2000).

### 3.14 *Kalanchoe pinnata* (Lam) Pers. [Crassulaceae]

*Kalanchoe pinnata* (Lam) Pers. is a medicinal plant that is mostly utilized in African, Brazilian, and Indian traditional medicine to treat a variety of human ailments (Bhavsar and Chandel, 2022). Fresh *Kalanchoe pinnata* (Lam.) Pers., leaf ethanol extracts are used to treat local skin lesions or taken orally for systemic effects (Fürer et al., 2016). Several active phytochemicals, such as flavonoids, glycosides, triterpenoids, steroids, bufadienolides, and organic acids have been identified in Kalanchoe pinnata (Lam.) Pers., leaves, which have been shown individually to possess a variety of biological and pharmacological activities (Prasad, 2012).

Ethanolic extract of fresh leaves of Kalanchoe pinnata (Lam.) Pers., induced a weak genotoxic response *in vivo* MN assay in mice. However, the extract did not induce reverse mutations in *Salmonella* as assayed by Ames test (Saravanan, et al., 2020). Phytol, oleic acid, flavone and methyl ester of octadecanoic acid were identified as the major phytochemicals in the ethanol extracts of fresh leaves Kalanchoe pinnata and some of these phytochemicals might be responsible for the genotoxic effect (Saravanan, et al., 2020). In another study, fresh Kalanchoe pinnata (Lam.) Pers. leaf juice was found to have no genotoxic effects (Bhavsar and Chandel, 2022). In the CBMN assay, the cytokinesis block proliferation index, cytotoxicity, micronuclei, nuclear bud, nucleoplasmic bridge frequencies, and total DNA damage biomarkers showed no significant changes for both 50 and 70 µL compared to the negative control. The changes observed were significant only at 70 µL for MI, which was significantly lower than that by positive control indicating a non-genotoxic effect. Based on the findings, the authors suggested that fresh leaf juice can be used both pharmaceutically and traditionally; however, it should be used with caution for long periods and in higher doses, as it can have mutagenic effects at particularly high levels (Bhavsar and Chandel, 2022).

### 3.15 *Nigella sativa* L. [Ranunculaceae]

*Nigella sativa* L., or black cumin belongs to the Ranunculaceae family, it is used as a spice and as a natural remedy against a great variety of illnesses (Al-Naqeep et al., 2011). The oil and seeds have shown potential medicinal properties in traditional medicine for several illnesses including asthma, cough, hypertension, bronchitis, diabetes, headache, eczema, fever, inflammations, and other diseases (Al-Naqeep et al., 2011). *Nigella sativa* oil, which contains the active ingredients thymoquinone and P-cymene, was reported to have anti-inflammatory and antioxidant properties (Al-Okbi et al., 2018). It has been demonstrated that *Nigella sativa* oil can protect against genotoxicity agents *in vitro*. The effects of *Nigella sativa oil* on MN formation in healthy human lymphocytes at concentrations of 1, 5, and 10 g/mL were investigated, and the genotoxic effects of CP used as a positive control were reduced by *Nigella sativa* oil in all tested concentrations (Zor and Aslan, 2020). In addition, Nguyen et al., 2019, has investigated *in vitro* cytotoxicity, genotoxicity and antigenotoxicity of aqueous seed extracts of *Nigella sativa* seeds originating from three different regions in Morocco using Ames assays, the MN and comet tests in human C3A cells. *Nigella sativa* seed extracts were shown to be not genotoxic against human C3A cells. Another study on the mixture of *Nigella sativa* seeds, *Hemidesmus* indicus roots, and *Smilax glabra* rhizomes was investigated on human lymphocytes stressed with bleomycin (Galhena et al., 2017). Moreover, *Nigella sativa* oil showed antigenotoxic effects against cisplatin-induced MN in polychromatic erythrocytes of BALB/c mice peripheral blood (Franco-Ramos et al., 2020). The combination could protect the cells against cytogenetic damage caused by bleomycin in human peripheral lymphocytes. In addition, Abdel-Moneim et al. (2017), indicated that *Nigella sativa* was efficient in the protection of CCl4-induced genetic damage in germ cells and could be utilized as a supplementary nutritional supplement in the early phases of mutagen exposure.

### 3.16 Gratiola officinalis L. [Plantaginaceae]

*Gratiola officinalis* L., belongs to Plantaginaceae family, and it has been used to cure liver ailments, visceral blockages, skin diseases, menstrual disorders, gout, and parasitic worms (Slapsytė et al., 2019). *Gratiola officinalis* L., extract has been shown to have anticancer and apoptotic activities (Navolokin et al., 2023), anti-inflammatory, antipyretic and antimicrobial activity as well as lipid hydroperoxide reduction (Navolokin et al., 2017). Slapsytė et al. (2019), reported that the methanol and acetone extracts from the whole plant of *Gratiola officinalis* L. induced MN and induced significant changes in NDI in human lymphocytes cells, indicating possible genotoxic effect at 37.5 μg/mL as assayed using CBMN assay (Slapsytė et al., 2019). In addition, the extracts, using Ames test *Gratiola officinalis* L., at 100 μg/plate without metabolic activation produced a 2-fold increase in the mean revertants per plate in TA98 strain showing that mutagenic effect at this concentration. Using Comet assay, *Gratiola officinalis* L., extracts induced DNA damage in a dose-dependent manner (Slapsytė et al., 2019). On the other hand, the antimutagenic effect of the flavonoids extracted from the Gratiola *officinalis* plant was reported in the literature. Durnova & Kurchatova, (2015) found that the flavonoids extracted from *Gratiola officinalis* reduced the mutagenic action of genotoxic agent CP in peripheral blood erythrocytes from mice at a dose of 200 mg/kg by reducing the number of MN. The genotoxic and antigenotoxic effects produced by the *Gratiola officinalis* L., might be due to the different extract and assay applied in the two studies. Phytochemical analysis showed that verbascoside was one of the most potent phenylporpanoids found in *Gratiola officinalis* L., plant extract (Slapsytė et al., 2019), in another study, verbascoside was found to induce genotoxicity in human lymphocytes, (Santoro et al., 2008). On the other hand, verbascoside did not show any mutagenic effects on chromosomes in *in vivo* studies with rabbits, including chromosome abnormalities, such as breaks and sister chromatid exchange (Perucatti et al., 2018). As the author suggested, the molecule that caused the genotoxic effects of *Gratiola officinalis* L., extract needs to be identified.

### 3.17 *Olea europaea* L. [Oleaceae]

Olive leaf extract (*Olea europaea* L.) from the Oleaceae family is a polyphenol-rich extract that contains polyphenols in substantially higher concentrations than extra virgin olive oil (Silva et al., 2006). Dried leaf is used to treat diarrhea and respiratory and urinary tract infections (Bellakhdar et al., 1991), anti-inflammatory and anticancer properties (Goldsmith et al., 2015). Verschaeve et al. (2017) studied the *in vitro* genotoxicity of the aqueous leaf extracts from four varieties of *Olea europea* L., from different regions in Tunisia using the alkaline comet assay on human C3A hepatic cells. None of the extracts were found to be genotoxic. Numerous *in vitro* and *in vivo* investigations have demonstrated the antiatherogenic, anti-inflammatory, anticancerogenic, and strong antioxidant activities of this olive leaf extract and its components (Čabarkapa et al., 2017). Topalović et al. (2019), used the comet test to study the antigenotoxic potential of a standardized methanol dry olive leaf extract on DNA damage induced by two estrogenic chemicals, 17-estradiol and diethylstilbestrol, in human whole blood cells. At the tested concentrations (up to 1 mg/mL) and under two experimental protocols, pre-treatment and post-treatment, dry olive leaf extract was effective in reducing the number of cells with estrogen-induced DNA damage, exhibiting antigenotoxic effect. The protective capability of dry olive leaf extract stemmed from the synergistic effect of its scavenging activity and enhancement of the cells’ antioxidant capacity of the cells. Secoiridoid oleuropein, triterpenes, flavonoids, caffeic acid and tannins might be responsible phytochemicals for this effect (Topalović et al., 2019). In addition, dry olive leaf extract was shown to reduce DNA damage induced by H2O2 and L-thyroxine-oxidizing agents in human peripheral blood leukocytes (Topalović et al., 2015), demonstrating a protective action against L-thyroxine effect. The anti-genotoxic effect was explained by its ability to serve as a powerful free radical scavenger. Furthermore, a study using a commercial dry olive leaf extract showed that DNA was protected from the genotoxic effects of adrenaline and hydrogen peroxide in human leukocytes as assayed by comet assay (Cabarkapa et al., 2014). The combinatorial activation of multiple molecular processes, including ROS scavenging and enhancing cellular antioxidant capacity, was likely the cause of dry olive leaf extract protective activity.

### 3.18 *Pterolobium stellatum* (Forssk.) Brenan [Fabaceae]

*Pterolobium stellatum* (Forsk.) Brenan., belongs to the Fabaceae family and is widespread in Africa. Fresh leaves and roots are chewed for medicinal purposes for tuberculosis and related respiratory diseases, diarrhorea, epilepsy and neuralgia (Andualem et al., 2014). Boiled roots are also used to treat common colds, persistent cough (asthma), and splenomegaly (Kigen et al., 2016). Chloroform and methanol extracts from *Pterolobium stellatum* (Forsk.) Brenan., roots caused DNA damage and increased tail DNA percentage in a concentration dependent manner as assayed by comet assay (Kahaliw et al., 2018). Phytochemical screening of the ethanolic extract of *Pterolobium stellatum* (Forsk.) Brenan., root revealed the presence of phenols, flavonoids, glycosides, tannins, saponins, and terpenoids (Teshome et al., 2023). More research is needed to identify the principal genotoxic compounds in the *Pterolobium stellatum* (Forsk.) Brenan., root.

### 3.19 *Dracocephalum moldavica* L. [Lamiaceae]

European *Dracocephalum moldavica* L., is one of the Lamiaceae family members, and has traditionally been used to treat stomach, hepatic, and cardiovascular diseases, as well as headache (Dastmalchi et al., 2007). According to Yang et al. (2014), the aerial parts of the plant contain a variety of polyphenols, particularly hydroxycinnamic acids (rosmarinic and caffeic acids) and flavonoids such as apigenin, luteolin and its glycosides, quercetin, diosmetin, kaempferol, acacetin, agastachioside, and salvigenin. *Dracocephalum moldavica* L., extract was reported to possess cardioprotective and antiplatelet (Jiang et al., 2014). Aprotosoaie et al. (2016) evaluated the antigenotoxic effect of a crude hydromethanolic extract of Dracocephalum moldavica L., aerial parts on normal human dermal fibroblasts stimulated with the genotoxic agent bleomycin which was known to cause single strand DNA breaks double strand DNA breaks and even clustered DNA lesions (Aziz et al., 2012). The antigenotoxicity was assessed using the *in vitro* comet test and the CBMN assay. As demonstrated by the comet assay, Dracocephalum moldavica L., extract treatment significantly reduced bleomycin-induced DNA damage in a concentration-dependent manner as compared to cells treated with bleomycin alone. The total number of MN in normal human dermal fibroblasts was raised by bleomycin; however, MN induction significantly decreased when fibroblasts were treated with extract from Dracocephalum moldavica L., at concentrations of 25 and 100 μg/mL (Aprotosoaie et al., 2016). Several polyphenols were identified in Dracocephalum moldavica L., extract including quercetin, apigenin 7-O-glucoside, rosmarinic acid, and chlorogenic and caffeic acids, being rosmarinic acid was the main polyphenol (Aprotosoaie et al., 2016). Dracocephalum moldavica L., extract exerted its protective effects against bleomycin-induced DNA damage and MN by its iron-chelating qualities, free radical scavenging activity, and the possible intervention on DNA repair processes due to the synergistic action of polyphenols metabolites presented in the extract mainly rosmarinic acid (Aprotosoaie et al., 2016).

### 3.20 *Solanum lycocarpum* A.St.-Hil. [Solanaceae]

*Solanum lycocarpum* A. St.-Hil*.,* is a member of the Solanaceae family, and a native Brazilian plant that is utilized in traditional medicine (Oliveira et al., 2003). The green fruits are topically applied on snake bites, while the hot baked fruit is utilized to alleviate tissue atrophy (Dall’ agnol and Lino van Poser, 2000). *Solanum lycocarpum* A. St.-Hil., is known for their high alkaloidic concentration and solasonine and solamargine are two major glycoalkaloids found in *Solanum lycocarpum* A. St.-Hil., (Tavares et al., 2011).

**FIGURE 4:**
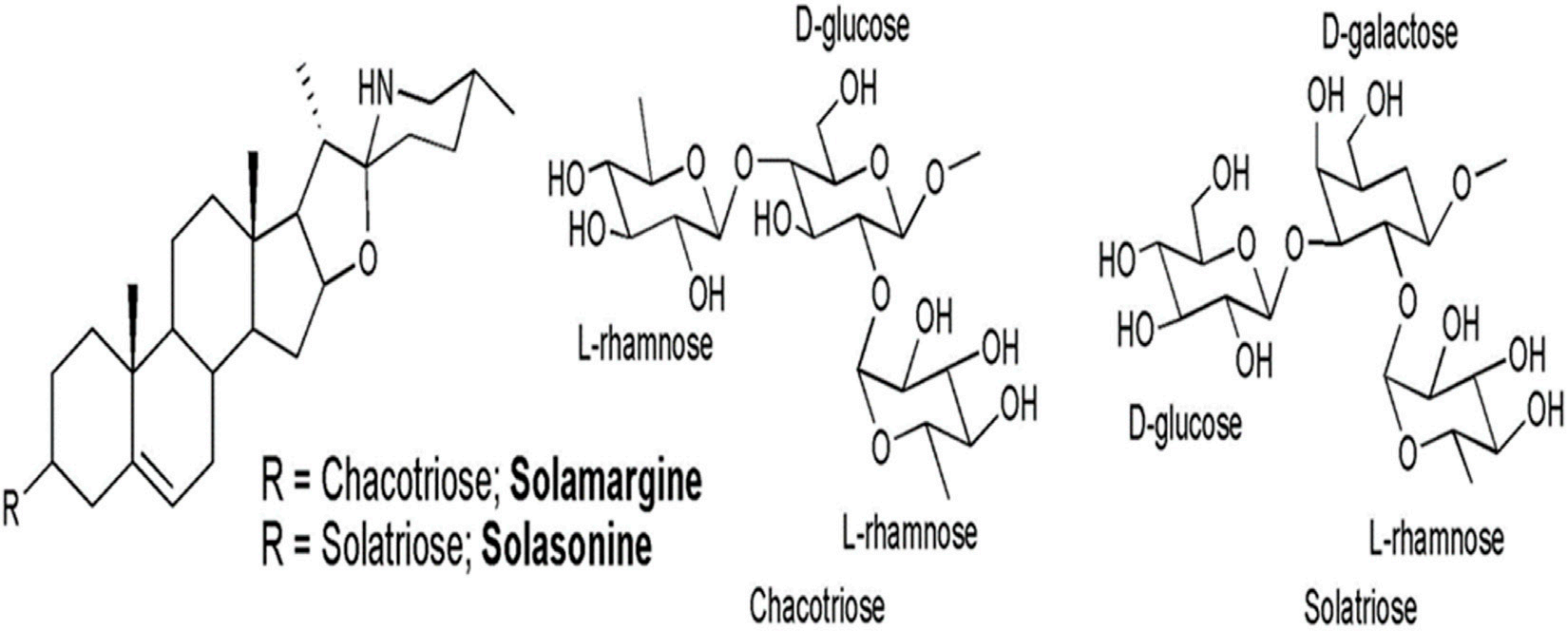
Chemical structures of solasonine and solamargine, the two main glycoalkaloids of *Solanum lycocarpum* fruits hydroalcoholic extract (Andrade et al. (2016).

Andrade et al. (2016) investigated the antigenotoxic potential of hydroalcoholic extract of *Solanum lycocarpum* A. St.-Hil., fruits using V79 cells. DNA damage was induced using well-known genotoxic chemicals Methyl methanesulfonate (MMS). *In vitro* comet and chromosomal abnormalities assays were used to assess genotoxicity in V79 cells. In comparison to cells treated with MMS alone, *Solanum lycocarpum* A. St.-Hil., fruits extracted at 8, 16, or 32 g/mL significantly reduced DNA damage and the frequency of chromosomal aberrations in V79 cells (Andrade et al. 2016). Chemical analysis of the hydroalcoholic extract of *Solanum lycocarpum* A. St.-Hil., fruits confirmed the presence of glycoalkaloids, solamargine and solasonine as the main secondary metabolites in *Solanum lycocarpum A. St.-Hil.,* fruit extract. The antigenotoxic effect of Solanum lycocarpum fruits glycoalkaloid extract might be due to the sum of interactions between different phytochemicals of this complex mixture which contains glycoalkaloids and phenolic compounds as the major secondary metabolites (Andrade et al., 2016). In addition, Tavares et al. (2011) reported similar outcomes with the hydroalcoholic extract of *Solanum lycocarpum* A. St.-Hil., fruits, which decreased the chromosomal damage in V79 cells caused by doxorubicin. The protective effect of *Solanum lycocarpum* A. St.-Hil., fruits glycoalkaloid extract can be attributed to its major glycoalkaloids, solamargine and solasonine (Figure 4), contributing to the reduction of DNA damage induced by MMS. According to Weissenberg, (2001) solasodine was found to be effective in the repair of DNA fragments.

### 3.21 *Euphorbia hyssopifolia* L. [Euphorbiaceae]

*Euphorbia hyssopifolia* L., belongs to the Euphorbiaceae family and it has been used in traditional Indian, Asian, and Latin American medicine (Kane et al., 2009). The aerial part of the plant is used as tea for colds, stomach, and back problems, and it is also used as a tonic (Kane et al., 2009). Alkaline comet test and CBMN were used to determine the genotoxic effects of ethanolic extract of the aerial parts of the *Euphorbia hyssopifolia* L., comprising stem, leaves, and inflorescences in human HepG2 cell (Araújo et al., 2015). Genotoxic effects were shown at 0.1 and 1.0 mg/mL in the comet assay. Additionally, the 1.0 mg/mL concentration induced severe cell damage leading to cell death in the CBMN assay, indicating a cytotoxic effect for this concentration in the latter method.

The presence of mono and sesquiterpenes, triterpenes and steroids, saponnins, flavonoids, cynnamic derivatives, and hydrolysable tannins was shown by phytochemical screening, with flavonoids being the most abundant in the *Euphorbia hyssopifolia* L., extract (Araújo et al., 2015). Secondary metabolites discovered in various *Euphorbia* species L., have been linked to genotoxic action in the literature and the genotoxic effect was most likely due to the presence of terpenoids, which was already reported by Miyata et al. (2006). Flavonoids, on the other hand, may be linked to genotoxic effects depending on the dose utilized, according to Soares et al. (2006). The genotoxic effect of *Euphorbia hyssopifolia* L., extract in HepG2 cells was explained to be due to the presence of mono and sesquiterpenes, triterpenes (Araújo et al., 2015).

### 3.22 *Salvia officinalis* L. [Lamiaceae]

*Salvia officinalis* L. (sage) from Lamiaceae family is a Mediterranean-native perennial woody subshrub. *Salvia officinalis* L., possesses antihydrotic, spasmolytic, antibacterial, and anti-inflammatory qualities and has been demonstrated to help cure mental and nervous problems (Baričevič and Bartol, 2000). This plant has also been reported as a potential treatment for cancer (Ho et al., 2000). Essential oil from leaves of *Salvia officinalis* L., was found to be safe and nongenotoxic when tested in mice bone marrow cells using comet assay and chromosomal aberrations test (Diab et al., 2018). In addition, the essential oil demonstrated the anti-genotoxic effect of carbon tetrachloride (CCl4) in mouse bone marrow and male germ cells. The essential oil from *Salvia officinalis* leaves contains monoterpenes, primarily eucalyptol, caryophyllene, β-pinene, and α-pinene (Diab et al., 2018). The authors suggested that the antigenotoxic effect of the *Salvia officinalis* L., essential oil might be through inhibition of the DNA-inducing agents before their attacking to DNA molecule, and the effect might be due to the presence of the monoterpenesphytochemicals (Diab et al., 2018).

**FIGURE 5:**
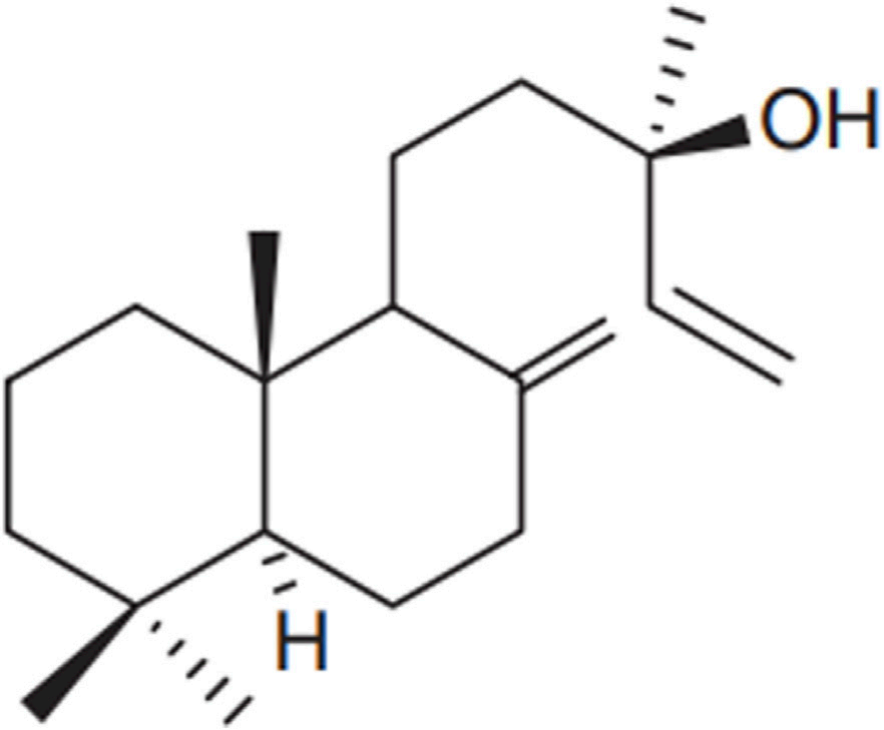
Chemical structure of the diterpene manool, Nicolella et al. (2014).

Diterpenes are the most distinctive metabolites of *Salvia* species, with manool (Figure 5) being the most abundant phytochemicals of *Salvia officinalis* L., essential oils and extracts (Velikovi et al., 2003). Manool has recently been proven to be effective against numerous periodontitis-causing bacteria (Souza et al., 2011).

Nicolella et al. (2014), used the CBMN to determine the genotoxic, and antigenotoxic potential of manool in V79 cells and HepG2. At the highest concentration tested, manool was genotoxic in both V79 (6.0 μg/mL) and HepG2 (8.0 μg/mL) cells. On the other hand, manool exhibited a protective effect against chromosome damage induced by methyl methanesulfonate (MMS) in HepG2 cells, but not in V79 cells. These findings suggested that some manool metabolite may be responsible for the antigenotoxic effect observed in HepG2 cells. The antigenotoxic effect of manool appears to be dependent on the concentration and the genotoxic model used. Manool at 0.5 and 1.0 μg/mL significantly reduced MN caused by doxorubicin (DOX) and hydrogen peroxide in V79 cells but did not affect etoposide-induced genotoxicity (Nicolella et al., 2021). A significant reduction in DOX-induced chromosomal damage was also seen in mice treated with 1.25 mg/kg; however, higher doses of manool (5.0 and 20 mg/kg) had no effect on the genotoxicity induced by doxorubicin (Nicolella et al., 2021).

### 3.23 *Cynara scolymus* L. [Asteraceae]

*Cynara scolymus* L., also known as artichoke, is a Mediterranean edible vegetable belonging to the Asteraceae family (Jacociunas et al., 2013). It is grown all over the world, and its leaves have been used as a choleretic and diuretic in traditional medicine since ancient times (Speroni et al., 2003). *Cynara scolymus* L., is a good source of natural antioxidants such as vitamin C, hydroxycinnamic acids, and flavones are abundant in it (Gil-Izquierdo et al., 2001). Aqueous extract of *Cynara scolymus* L., leaves showed a significant increase in MN frequencies, which represent chromosome breakage after 1 h and 24 h exposure to different concentrations of *Cynara scolymus* L., up to 5 mg/mL in CHO k1 cells using the CBMN assay (Jacociunas et al., 2013). High concentrations of *Cynara scolymus* L., leaves can pose a risk associated with its consumption. The increased MN frequencies in a dose-dependent manner, by the Cynara *scolymus* L., leaves extract might be a result of the pro-oxidant activity of the extract phytochemicals such as flavonoids and chlorogenic acids (Jacociunas et al., 2013). In contrast, Cynara scolymus L., leaf aqueous extract at concentrations up to 2000 mg/mL did not exhibit genotoxic or mutagenic activity *in vivo* when tested on mice peripheral blood cells using the MN test. However, a significant increase in comet assay value in mice bone marrow treated with 2000 mg/kg was observed. The highest dose studied suggested that Cynara scolymus L., tea should be consumed in moderation (Zan et al., 2013). da Silva et al. (2017) revealed that Cynara scolymus L., leaf aqueous extract had a genotoxic effect on HepG2 cells as determined by the comet test at 4 extract concentrations (0.62, 1.25, 2.5, and 5.0 mg/mL). Nevertheless, the lowest concentration (0.62 mg/mL) reduced the incidence of DNA damage caused by H_2_O_2_.

The main active phytochemicals found in *Cynara scolymus* L., leaves extract were flavonoids, phenolic metabolites, and saponins. Among these phytochemicals, isoquercitrin, followed by chlorogenic acid were the main metabolites. Caffeic acid and ferulic acid are two phenolic acids described as leaves constituents, along with flavonoids (Azzini et al., 2007). Furthermore, luteolin and luteolin glycosides have been found in the *Cynara scolymus* L., leaves extract (Fratianni et al., 2007). As previously suggested (Miadokova et al., 2008), the oxidation of such compounds as catechol, a structural element metabolites of many flavonoids presented in *Cynara scolymus* L., leaves extract, like the quinones quercetin and luteolin and their isomeric quinone methides, generated electrophilic compounds that could alkylate DNA.

### 3.24 *Dendrobium speciosum* Sm. [Orchidaceaeis]

*Dendrobium* species have been found to produce alkaloids and bibenzyl phytochemicals, as well as phenanthrenes and stilbenes (Li et al., 2009). *Dendrobium speciosum* Sm., belongs to Orchidaceaeis family, a widely widespread *Dendrobium* species in Australia, whose starchy stems are eaten raw or roasted (The Royal Botanic Gardens and Domain Trust, 2013). Moretti et al. (2013) investigated the antigenotoxicity of *Dendrobium speciosum* Sm., Leaves and stems methanolic extracts on DNA damage induced by nitroquinoline N-oxide in HepG2 cells. Antigenotoxicity was assayed using a comet assay. Methanolic extracts of *Dendrobium speciosum* Sm., stems reduced the degree of DNA damage caused by nitroquinoline N-oxide (4-NQO) in HepG2 cells at low doses of 2.5, 5.0, and 10.0 μg/mL when compared to the negative control. On the other hand, the leaf extract caused a substantial increase in the level of DNA damage at the higher concentration tested (100 μg/mL) (Moretti et al., 2013). Chemical analysis showed that the stem extract contained palmitic, oleic and linoleic acid with a balanced ratio of linoleic to α-linolenic acid. The methanolic extract of leaves and stems contains polyphenols and flavonoids (Moretti et al., 2013).

### 3.25 Bauhinia platypetala Burch. ex Benth. [Bauhinia]

*Bauhinia platypetala* Burch ex Benth., belonging to the Bauhinia family, is a popular diabetes treatment in Brazil (Vaz and Tozzi, 2005). The genotoxic potential of the ethanolic extract and its ethereal fraction from leaves in V79 cells was reported (Santos et al., 2012). The ethanolic extract and its ethereal fraction induced DNA damage measured by increased damage index and damage frequency in V79 cells as indicated by comet assay. Hexadecanoic acid, kaempferitirin, and quercitrin were the main phytochemicals in the *Bauhinia platypetala* Burch ex Benth., ethanolic leaves extract, while phytol, gamma-sitosterol, and vitamin E were abundant in the *Bauhinia platypetala* Burch ex Benth., ethereal fraction (Santos et al., 2012). The DNA damage induced by the ethanolic extract and its ethereal fraction might be due to the presence of several phytochemicals, such as hexadecenoic acid (Santos et al., 2012). Hexadecenoic acid was reported to induce DNA damage and cause apoptotic cell death in insulin-producing beta-cells (Beeharry et al., 2004; Sanz-Serrano et al., 2021). Nevertheless, more studies are needed to determine the responsible phytochemicals and the molecular mechanism underlying *Bauhinia platypetala Burch ex Benth.,* -induced genotoxicity.

### 3.26 *Punica granatum* L. [Lythraceae]

*Punica granatum* L., also known as pomegranate, is a member of the Lythraceae family and has been utilized in medicine to treat some diseases (Jurenka, 2008). *Punica granatum* L., leaves have been shown to be anti-obesity (Lei et al., 2007) and improve cerebral blood flow and heal skin ulcers (Xiang et al., 2008). Dassprakash et al. (2012) studied the effect of aqueous extract of *Punica granatum* L., leaves on cyclophosphamide-induced genotoxicity in mouse bone marrow cells using MN test. Aqueous extract of *Punica granatum* L., leaves was effective in exerting significant antigenotoxic effects by reducing micronucleated polychromatic erythrocytes cells and total percentage of polychromatic erythrocytes in bone marrow cells of treated mice compared to untreated control mice at tested concentrations of 400, 600, and 800 mg/kg body weight. *Punica granatum* l., leaves phytochemical examination revealed the presence of flavonoids, phenols, phytosterols, tannins, and carbohydrates. The antigenotoxic protection against cyclophosphamide-induced genotoxicity seen in *Punica granatum* l., leaves extract might be due to the presence of these phytochemicals which may interact synergistically or additively to exert the anti-genotoxic effect through antioxidant effects (Dassprakash et al., 2012).

### 3.27 *Hemidesmus indicus* (L.) R. Br. [Apocynaceae]

*Hemidesmus indicus* Linn. R. Br., is one of the Asclepiadaceae family member which has been widely utilized in the treatment of a variety of ailments such as leprosy, leucoderma, leucorrhoea, syphilis, rheumatism, asthma, and bronchitis (Ananthi et al., 2010). Several pharmacological properties have been reported for Hemidesmus indicus Linn. R. Br., root extracts, including pregnane glycosides, tannins, saponins, resin acid, lupeol acetate, -sitosterol, amyrins, 2-hydroxy-4-methoxy-benzoic acid, and triterpenes (George et al., 2008).

According to Ananthi et al. (2010), the methanolic extract of *Hemidesmus indicus* L. R. Br., root showed a significant increase in the percentage of chromosome aberrations at the higher concentration of 32 μg/mL. The presence of saponins, tannins, flavonoids, alkaloids, phenols, coumarins, and terpenoids in the ethanolic extract of *Hemidesmus indicus* L. R. Br., roots might be the responsible phytochemicals for the genotoxic effect (Ananthi et al., 2010). These substances have been shown to have the potential to cause the development of MN in several investigations. On the other hand, Hemidesmus indicus root extract has a significant antigenotoxic effect against cisplatin-induced frequencies of micronucleated binucleated cells at the lower concentrations of 4 and 8 µg/mL. The antigenotoxic effect could be attributed to the protective antioxidant effect of its constituents as medicinal herbs contain complex mixtures of several phytochemicals that can act singly or synergistically. The bioactive compound extracted from the roots of *Hemidesmus indicus* L. R. Br., indicus, 2-hydroxy-4-methoxy benzoic acid and the terpenoidal fraction of the root bark demonstrated to scavenge free radicals (Kumar et al., 2008). Shetty et al. (2005) reported that the methanolic extract of *Hemidesmus indicus* L. R. Br., root protects plasmid DNA from radiation-induced strand breakage as well as rat liver microsomal membranes from lipid peroxidation.

### 3.28 *Phyllanthus emblica* L. [Phyllanthaceae]

*Phyllanthus emblica* L., a member of the Euphorbiaceae family, is a fruit-bearing plant found in tropical and subtropical regions of India, China, Thailand, Indonesia, and the Malay Peninsula (Guo, et al., 2017a). The fruit of *Phyllanthus emblica* L., is widely ingested as a functional food and employed as a traditional remedy due to its exceptional nutritional and pharmacological effects (Prananda et al., 2023). Various parts of *Phyllanthus emblica* L., plants, such as the fruit, flower, seed, leaf, root, and bark, have been extensively utilized in diverse Asian traditional medicinal systems for millennia (Guo X. H. et al., 2017).

Through phytochemical research, numerous categories of metabolites like flavonoids, glycosides, terpenoids, and phenolic phytochemicals have been identified in this plant (Zhang et al., 2016). Via fraction-based fractionation, chemical compounds such as gallic acid, phyllemblin, corilagin, furosin, and geranin have been previously documented (Liu et al., 2008). Additionally*, Phyllanthus emblica* L., fruits contain flavonoids like quercetin and alkaloids such as phyllantine and phyllantidine. Several biological activities of this plant were reported including antioxidant, anti-aging, anti-cholesterol,anti-diabetic, immunomodulatory, antipyretic, analgesic, anti-inflammatory, chemoprotective, hepatoprotective, cardioprotective, antimutagenic, and antimicrobial properties (Prananda et al., 2023; Saini et al., 2022).

Several research studies have confirmed the safety of *Phyllanthus emblica* L., both in in vivo and *in vitro*. A previous investigation found no evidence of toxicity from *Phyllanthus emblica* L., fruit extract at doses up to 500 mg/kg (Anto et al., 2022). Additionally, it was found that the ethanolic extract of *Phyllanthus emblica* L., is safe for rats at doses of up to 2000 mg/kg (Uddin et al., 2016). Chronic toxicity studies involving oral doses of *Phyllanthus emblica* L., up to 1,200 mg/kg for 270 days showed no pathological changes in treated animals (Jaijoy et al., 2010).

Regarding genotoxicity studies, it was demonstrated that the *Phyllanthus emblica* L., plant exhibits antigenotoxic properties. Specifically, the aqueous extract of *Phyllanthus emblica* L., fruits was observed to enhance the effectiveness of MMC and cisplatin (cDDP) in NCM460 normal colonic cells, while also playing a significant role in reducing the frequencies of MN. This resulted in genome protection from instability and inhibition of the clonal expansion of an unstable genome induced by MMC and cDDP in NCM460 normal colonic cells, as determined by CBMN assay. This response was attributed to the antioxidant characteristics of the aqueous extract of *Phyllanthus emblica* L., fruits (Guo X. H. et al., 2017). Furthermore, it was found that the aqueous extract of *Phyllanthus emblica* L*.,* exhibited antigenotoxic effects by preventing mitosis exit through activation of the spindle assembly checkpoint in human NCM460 normal colon epithelial cells (Guo and Wang, 2016).

**FIGURE 6:**
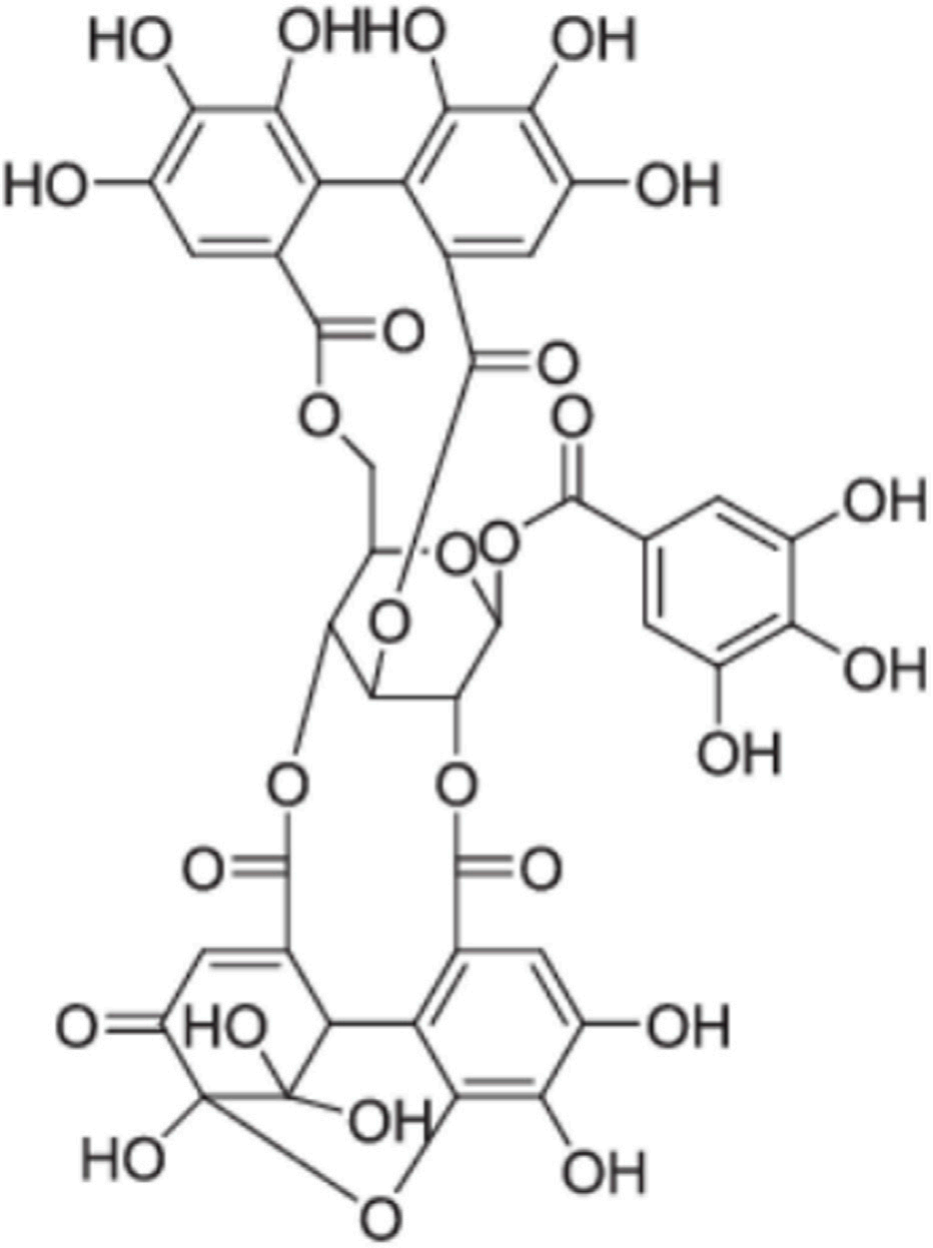
Chemical structures of geraniin, Guo et al. (2018).

The impact of aqueous fruit extracts of *Phyllanthus emblica* L., fruits extract on genomic damage and cell death in the human colon adenocarcinoma cell line COLO320 was investigated in another study. The study utilized the cytokinesis-block micronucleus assay and found that the aqueous fruit extracts suppressed necrosis and had a dose- and time-dependent genotoxic effect on COLO320 cells. This was evidenced by an increase in the frequencies of MN, nucleoplasmic bridges, and nuclear buds (NBuds) in COLO320 cells, as reported by Guo et al. (2013). These biomarkers of DNA damage offer a reliable measure of chromosomal instability. The plant extract’s efficacy was believed to be linked to its active, geraniin (Figure 6). According to a different study, geraniin specifically encourages cytostasis and apoptosis in human colorectal cancer cells by triggering severe chromosomal instability (Guo et al., 2018). It was found that geraniin significantly raised the frequency of MN and NPB in Colo320 cells in a manner dependent on both dosage and time, indicating its ability to induce chromosomal instability in cancer cells. Nevertheless, in normal cells, NCM460, a dose- and time-dependent decrease in in the frequencies of MN, nucleoplasmic bridges, and nuclear buds was observed following geraniin treatment, suggesting an antigenotoxic effect (Guo et al., 2018) in comparison to the control group.

A summary of the selected studies including, plant name/family, part used for extraction, concentration, genotoxicity assay, model organism/cell line used, positive control used, effect and reported mechanisms are presented in Tables 1–3.

## 4 Discussion

### 4.1 Genotoxicity assays, plant parts used and extraction

**FIGURE 7:**
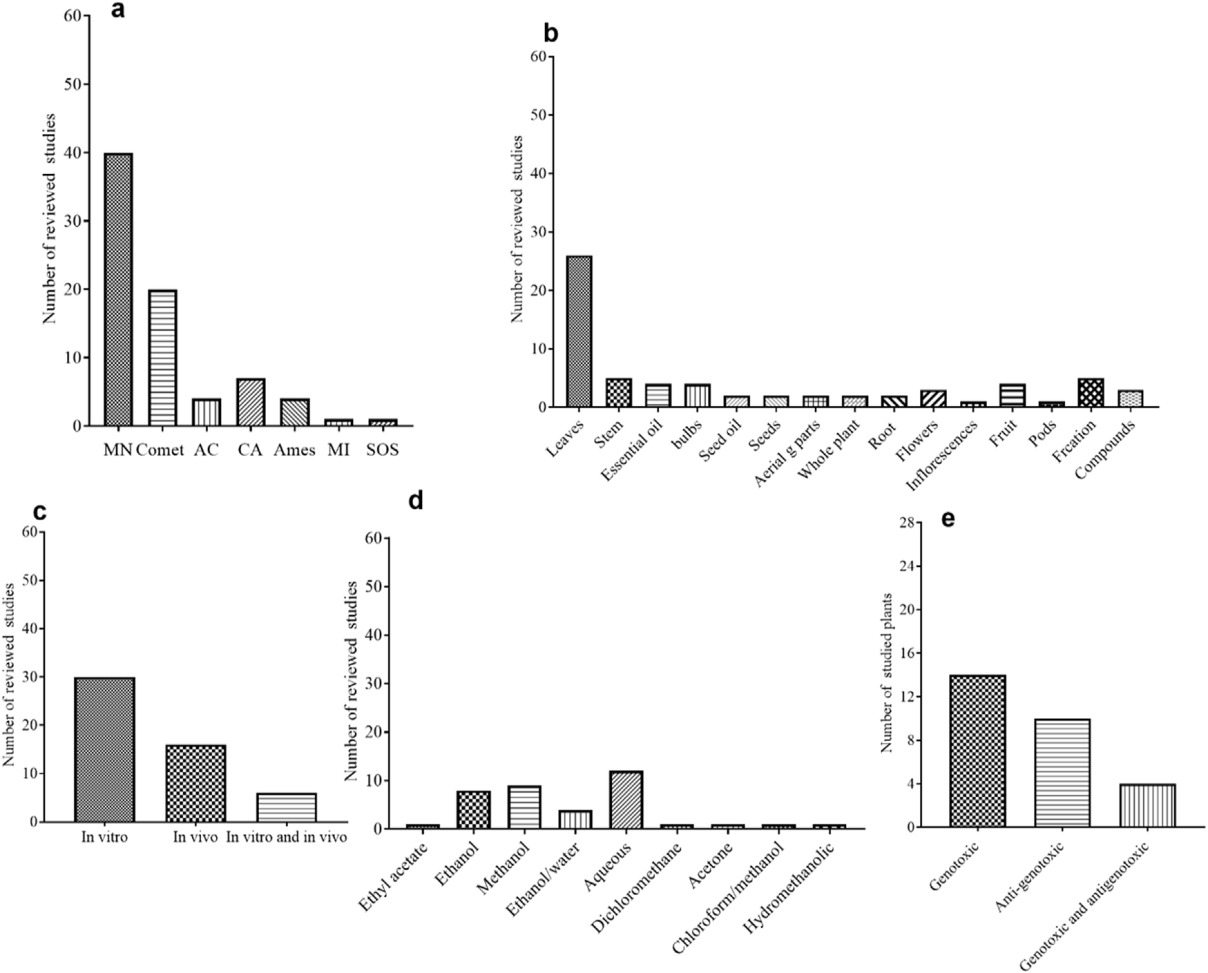
**(A)** Genotoxicity assays used in selected plants extract studies, as calculated from 52 reviewed articles. MN = Micronuclei assay, CA = Chromosomal aberration, MI = Mitotic Index, the AC = Allium cepa test, the Ames = *Salmonella*/microsome or bacterial reverse mutation assay, SOS = the SOS Induc test. **(B)** *In vitro*, *in vivo* and both *in vitro* and *in vivo* genotoxicity assays used in selected plant extracts studies, as calculated from 52 reviewed articles. **(C)** Part of plant used in the extraction for genotoxicity studies, as calculated from 52 reviewed articles. **(D)** Solvents used for extraction for genotoxicity studies used in selected plants extract, as calculated from 52 reviewed articles and **(E)** Different genotoxic effects of selected plant extracts, as calculated from 52 reviewed articles.

In this review, the genotoxicity studies of some selected plant extracts were determined using various assays, including *in vivo* or *in vitro* MN, comet assay, Ames test, Allium cepa test, and chromosomal aberration assay. In some studies, more than one test was used to assess the genotoxicity of a specific plant extract. The authors most frequently utilized the MN assay, followed by comet assays as shown in Figure 7A. Several studies that are discussed in this review used *in vitro* assays and some studies were reported using *in vivo* or both *in vitro* and *in vivo* assays (Figure 7B). Different models in genotoxicity studies were used mainly *in vitro* cell lines including, HepG2, HepG2/C3A, PDLSCs, human lymphocytes cells, CHO–K1, NCM460, peripheral blood cell, MNP01 and V79 cells. *In vivo* genotoxicity assay has been utilized to verify *in vitro* assay results and certainly provide biological significance for certain organs or cell types (Kang et al., 2013). Most of the reviewed studies were based on *in vivo* comet and MN assays. Bone marrow of Wistar rats, peripheral blood cells of rats and mice, liver and brain tissues of rats and onion bulbs were used as models for *in vivo* assay in the reviewed articles.

Preparation of the extract from the desired parts of the plant is a major determining step in any scientific study. The extraction from different parts of the plants including leaves, whole plant, flower, steam, bulbs, roots, seeds, seeds oil, essential oils and fresh pods were applied for the genotoxicity studies in the reviewed articles, being the extraction from leaves parts are shown to be the most used part (Figure 7C). The reviewed articles included various extraction methods such as maceration, digestion, infusion, and Soxhlet extraction. Of these methods, the maceration process was found to be the most frequently utilized. Water, Ethanol and methanol were the most used solvents (Figure 7D). The review covered an aqueous extract of 12 plants, and 7 of them showed genotoxic effects from different parts of plants.

### 4.2 Genotoxicity findings and the influence of experimental conditions

Most of the reviewed articles investigated the genotoxicity tests utilizing crude plant extracts. Crude plant extract may include hundreds of different biologically active phytochemicals in a variety of abundances, making it difficult to identify the bioactive phytochemical responsible for a specific biological action (Enke and Nagels, 2011). Indeed, the combined action of various phytochemicals with synergistic, additive, or antagonistic activity results in the overall activity of medicinal plant extracts (Junio et al., 2011). Based on the reviewed studies conditions, a total of 4 plant extracts showed no genotoxic effect, other 14 plant extracts showed either genotoxic or mutagenic effect and 14 plant extracts showed anti-genotoxic effect against several genotoxic induced agents. In addition, 4 plant extracts showed both genotoxic and antigenotoxic effects (Figure 7E).

Genotoxicity findings in some reviewed articles were dependent on experimental conditions including concentrations used, different assays used, the model organism/cell line used, different plant parts, extraction method and solvents used. Nevertheless, a wide range of concentrations or doses and more than one technique is critical to confirm genotoxicity findings (Al-Anizi et al., 2014). In one reported study with the same experimental conditions, the genotoxic finding differed based on the used concentrations. For instance, *Eugenia uniflora* L., extract showed a mutagenic effect at lower dose of 100 μg/plate in the Ames test when the extract was tested at a concentration range of 100–500 μg/plate. The same extract of *Eugenia uniflora* L., showed a genotoxic effect at only 37.5 μg/mL in CBMN when tested at a concentration range between 12.5 and 50 μg/mL (Slapsytė et al., 2019). In addition, the ethanolic extract of *Hemidesmus indicus* (L.) R. Br. roots showed a genotoxic effect at higher concentrations, but an antigenotoxic effect at lower concentrations (Ananthi et al., 2010). On the contrary, aqueous extract, and the oil from *Eugenia uniflora* L., showed a mutagenic effect at lower concentration tested, and at higher concentration was considered to have an antimutagenic effect (Kuhn et al., 2015). Moreover, manool from *Salvia officinal* L., dried leaves was also genotoxic at the highest dosage tested and exhibited a protective effect against chromosome damage at lower doses caused by MMS (Nicolella et al., 2014). Manool also showed an antigenotoxic effect at a lower dose (1.25 mg/kg b.) *in vivo* (Nicolella et al., 2021). At the same tested concentrations, some plant extracts exhibited both genotoxic and antigenotoxic phytochemicals effects like *Artemisia vulgaris* L., and *Artemisia alba* Turra., (Jakovljević et al., 2020). This can be explained by the fact that the plant extract contains complex mixtures of phytochemicals that may work synergistically, and while some phytochemicals in such plants are genotoxic, others are anti-genotoxic.

Within the same part of the plant, the *in vivo* genotoxicity findings differed based on the type of extraction solvent used, type of assay, concentrations used also the plant location or the lab where the assay was conducted. As it can be found in the ethanol/water extract of *Azadirachta indica* A. Juss., leaves showed in one study no-genotoxic effect *in vivo* MN test (Ramalho et al., 2023), and in another study, aqueous extract *of Azadirachta indica* A. Juss., leaves showed mutagenic effect *in vivo* Allium cepa assay. However, the 2 studies were conducted with different assays used, different solvent extracts, different concentrations and reported by different authors from different places. In the case of *Moringa oleifera* Lam., leave aqueous extract (Table 1) which show in one study to be non-genotoxic (de Barros et al., 2022) and in another 2 studies the aqueous extract was shown to have a genotoxic effect (Bakare et al., 2022; Asare et al., 2012). Again, the studies of *Moringa oleifera* Lam., leave aqueous extract were carried out *in vivo* with different assays, concentrations and different models used. The same trend of the findings was also found in *Eugenia uniflora* L., leave aqueous extract, in one study the extract was non-genotoxic in murine erythrocytes of the mouse (Ferreira et al., 2022) and in another study, it showed mutagenic activity in Onion bulbs (Kuhn et al., 2015). The 2 studies were conducted with different assays, different ranges of concentrations and reported by different Authors. In the case of *Gratiola officinalis* L., extracts, different extracts showed different genotoxicity findings even though the same *in vitro* assay was used, the methanol and acetone extracts from the whole plant showed a genotoxic effect (Slapsytė et al., 2019), however, the flavonoids extract from the *Gratiola officinalis* L., plant showed antimutagenic effect (Durnova and Kurchatova, 2015).

In some reviewed articles, the genotoxicity studies for some plant extracts were conducted both *in vitro and in vivo* using different experimental models. Sometimes, both *in vitro* and *in vivo* demonstrated the same genotoxicity response, even though different conditions were used. For instance, the anti-genotoxic effect of *Equisetum arvense* extracts and *Nigella sativa* seeds oil. In addition, to the genotoxic effect of *Eleutherine plicata* L., isolated phytochemicals (Table 1). In other cases, the opposite outcome was found within *in vivo* studies like the findings from *Azadirachta indica* A. Juss., extract. *Azadirachta indica* A. Juss., extract was positive genotoxic *in vivo* Allium cepa assay (Akaneme and Amaefule, 2012), but the effect was not confirmed in MN testing in rats (Ramalho et al., 2023). However, once again, the plant extracts in the two studies were prepared differently and reported by different Authors from different affiliations and countries. The same findings in the *Moringa oleifera* Lam., extract indicated positive genotoxic *in vivo* Allium cepa and chromosome aberration assays (Bakare et al., 2022; Asare et al., 2012). However, the findings were not confirmed with *in vivo* comet assay and MN test (de Barros et al., 2022). Once again, the assays and the concentrations used were different in these studies.

In the EMEA Guideline, for the genotoxicity assessment, it is recommended the following combination of tests: the Ames test, the mouse lymphoma assay (or other mammalian cell assays), and the rodent micronucleus test (or other *in vivo* genotoxicity tests) (Dantas et al., 2020). For the initial screening stage, for example, the EFSA Scientific Committee proposes a combination of two *in vitro* tests: the bacterial reverse mutation assay, which covers gene mutations, and the *in vitro* micronucleus test, which covers chromosome abnormalities. If *in vitro* testing finds positive genotoxic outcomes, additional *in vivo* testing is required. The EFSA recommends three *in vivo* tests: the mammalian erythrocyte micronucleus test, transgenic rodent somatic and germ cell gene mutation assays, and an *in vivo* comet assay (European Food Safety Authority, 2011). In the reviewed articles about 22 studies have reported the genotoxicity findings based on a single assay used either *in vitro*, which needs further investigations to confirm these findings. In addition, in some reviewed studies, a standard battery test that included the mutation test (Ames test) and genotoxicity in mammalian cells was applied and the findings from both tests were in agreement. For instance, both tests indicated the same genotoxicity response *Sapindus Saponaria* L. Only in one study of the *Kalanchoe pinnata* (Lam.) Pers., extract, Ames test yield negative results and the MN indicated slightly genotoxic effect.

Another factor that may have an impact on the findings is that most of the research analyzed was reported just once, which could indicate false positive or false negative findings that require assay validity. Nonetheless, examples of reproducibility of results were noticed when evaluating the same extract using the same techniques and concentrations, as observed for dry Olea europaea L., leaf extract. Another aspect of concern is that a plant’s characteristics can vary based on its growing environment. This is because they are exposed to different environmental conditions and thus respond differently to external stimuli (Zhou et al., 2013). Almost half of the extracts that showed genotoxic effects in the reviewed articles were from Brazil. Different conditions may also be caused by environmental contaminants, which play an important role in determining plant behaviors.

However, in the context of genotoxicity assessment, there is a double risk of false positive and false negative data. The assessment of toxicity and its level also impacts the outcomes of genotoxicity studies. Determining toxicity is crucial for selecting appropriate doses in genotoxicity evaluations. Prior to conducting genotoxicity testing, it is essential to determine the cytotoxicity of the plant extracts being tested and to select the suitable concentration range for subsequent genotoxicity assessments. In most cases, genotoxic substances are not recognized as such *in vitro* tests unless the concentrations being tested cause some level of cytotoxicity. Nevertheless, high levels of cytotoxicity can result in misleading positive or negative outcomes in genotoxicity evaluations. Instances of cell death can lead to double-strand DNA breaks that are not necessarily caused by primary or secondary genotoxicity (Elespuru et al., 2022). Furthermore, the preference for individual cytotoxicity tests during the assessment of genotoxicity is also emphasized by genotoxicity guidelines. The range of doses is contingent upon the specific genotoxicity test used. For example, in micronucleus assays, the concentration range must include non-cytotoxic levels as well as concentrations that result in approximately 50% ± 5% cell death (Kohl et al., 2020). When choosing a cytotoxicity test for use in genotoxicity assessments, it is crucial that the same exposure conditions are maintained for both tests, and that they are conducted concurrently (Efeoglu et al., 2018). For example, the OECD 487 (2010) recommends the use of a cytokinesis-block proliferation index (CBPI) or the Replication Index (RI) for cytotoxicity determination when utilizing the *in vitro* Cytokinesis-block micronucleus assay for genotoxicity determination. Evaluation of other cytotoxicity indicators (such as cell integrity, apoptosis, necrosis, metaphase counting, and cell cycle) may yield valuable information as part of the micronucleus experiment, which is more certain of the cytotoxicity of the tested concentrations. However, it should not be used in place of the RI or the CBPI, as recommended by the OECD guideline (OECD 487, 2010). Considering this, in some reviewed articles, the findings on the positive genotoxic effect of *Euphorbia hyssopifolia* L. (Bhavsar and Chandel. (2022); *Cynara scolymus* L., (Jacociunas et al., 2013), and *Smallanthus sonchifolius* (Poepp.) H. Rob., (Moreira Szokalo et al., 2020), using *in vitro* Cytokinesis-block micronucleus assay for genotoxicity determination, in these studies there was no mention of cytotoxicity determination using the indicators of cytotoxicity by CBPI or RI, even though some of these studies they reported using the cell micronucleus test recommended by OECD guide. Similarly, in the reported positive antigenotoxic effect of the *Dracocephalum moldavica* L., (Aprotosoaie et al., 2016), there was no cytotoxicity determination in the cells scored for MN using CBPI or RI. Hence cytotoxicity methodological might lead to the false positives. Based on the information gathered for this review, confirming the sensitivity, false positive, or false negative in these studies regarding the assessment of plant genotoxicity is quite challenging. These studies involved varying quantities and dosages, utilize different solvents, include extracts that are prepared in diverse ways, and sometimes examine different sections of the plants. Consequently, employing various methods to extract plant material results in the isolation of distinct chemical components, which may produce differing effects on toxicity and efficacy.

### 4.3 Anti-genotoxicity findings

For antigenotoxic findings in the reviewed articles (Table 2), the majority of the reviewed studies focused on examining the impact of plant extracts in conjunction with well-known DNA-damaging agents to determine their antigenotoxic properties. Various genotoxic chemical agents, including clastogens that do not require metabolic activation such as MMC, MMS, and NQO4, as well as clastogens that require metabolic activation like B [a]P and CP, were utilized as positive controls or to induce genotoxicity in different models across the reviewed articles. Additionally, clastogenic and aneugenic agents like DOX and compounds with aneugenic properties such as colchicine were also utilized. Some studies also employed agents like H_2_O_2_, catechol, bleomycin, cisplatin, and methotrexate. Certain review studies focused on using a single assay to study the antigenotoxic effect, without providing in-depth information on the mechanisms of action. For instance, an investigation into the essential oil extracted from *Croton blanchetianus* Baill., leaves revealed its ability to protect DNA against CP damage in mouse blood by reducing micronucleated polychromatic erythrocytes through the MN assay, without specifying the mechanism of DNA protection (Nascimento et al., 2024). Similarly, the antigenotoxic effect of the essential oils of *Hyssopus officinalis* L., against H_2_O_2_-induced DNA damage in human peripheral blood leukocytes was assessed using only one method which is comet assay (Mićović et al., 2021). Furthermore, the protective potential of *Nigella sativa L.,* seed oil and aqueous seed extracts against CP-induced MN production in human cells was evaluated solely through the comet test, without elaborating on the mechanism of action (Topalović et al., 2019). Additionally, the ability of extracts from *Artemisia vulgaris* L., and *Artemisia alba* Turra*.,* to protect against MMC-induced NDI in peripheral blood using CBMN was demonstrated, although the study exclusively utilized a single assay and did not explore the mechanism of action (Grujičić et al., 2020). Similarly, the assessment of the protective effect of *Nigella sativa* L*.,* seed oil and aqueous seed extracts against CP-induced MN formation in human lymphocytes was confined to the comet assay, with no reporting on the mechanism of action (ZOR and Aslan, 2020; Nguyen et al., 2019).

The authors in certain research studies discussed a proposed mechanism of action. For instance, they suggested that the antimutagenic effect of *Equisetum arvense* L., against CP *in vivo* might result from its antioxidant properties or its impact on DNA replication (Kour et al., 2017). The reported antigenotoxic effect of *Hyssopus officinalis* L., essential oils against H_2_O_2_-induced DNA damage in human peripheral blood leukocytes was linked to the neutralization of free radicals by the polyphenols present (Mićović et al., 2021).

Some plant extracts’ antigenotoxic effects were investigated using multiple assays *in vitro* and *in vivo* models. For example, the antigenotoxic effect of *Dracocephalum moldavica* L., was determined using the *in vitro* comet test and the CBMN assay. It was suggested that the protective effect against bleomycin-induced DNA damage and MN resulted from its iron-chelating qualities, free radical scavenging activity, and possible intervention in DNA repair processes due to the synergistic action of polyphenols phytochemicals, mainly rosmarinic acid (Aprotosoaie et al., 2016). Additionally, the hydroalcoholic extract of *Solanum lycocarpum* A.St.-Hil., fruits was evaluated for its ability to counteract the genotoxic effects of the MMS agent on V79 cells using both *in vitro* comet and chromosomal aberrations assays. The extract’s antigenotoxic properties were attributed to the main secondary metabolites, glycoalkaloids, and phenolic compounds (Andrade et al., 2016).

Upon review, most studies recommend further investigation to gain a better understanding of the principal antigenotoxic phytochemicals present in the extracts and the underlying mechanisms of antigenotoxicity. More extensive studies on these plant extracts are needed, as they may emerge as promising candidates for genoprotection.

### 4.4 Proposed phytochemicals responsible for genotoxicity response

Plant extracts contain phytochemicals that might be non-genotoxic, genotoxic or antigenotoxic. Among these phytochemicals are terpenes, which are secondary metabolites that are found in many plant families. Literature data indicated the genotoxicity and mutagenicity of monoterpene (Nesterkina et al., 2023; Baldissera et al., 2017). However, many monoterpenes are non-genotoxic and non-mutagenic in several biological test systems. Their activity may depend on the test concentrations/doses (Islam, 2017). In the reviewed articles, different classes of terpenes phytochemicals including mono and sesquiterpenes, diterpene phytol, sesquiterpene lactones were identified in different plants extracts that exerted genotoxic effect like *Smallanthus sonchifolius* (Poepp.) H. Rob., *Hemidesmus indicus* (L.) R. Br., *Pterolobium stellatum* (Forssk.) Brenan, *Kalanchoe pinnata* (Lam.) Pers., were suggested to be responsible for the genotoxic effect. On the other hand, different classes of terpenes phytochemicals were identified in the plant extracts that showed anti-genotoxic protection like *Croton blanchetianus* Baill., *Hyssopus officinalis* L., were proposed to be the responsible compounds for the anti-genotoxic effect.

Many plant species produce saponins, a wide class of bioactive compounds (Arslan and Ili, 2015). There have been reports of the genotoxic effects of saponin phytochemicals from various plant extracts (Demma et al., 2013). It was demonstrated that a mixture of saponins derived from Nauclea species caused synergistic *in vitro* chromosome mutations and DNA damage in mammalian cells (Liu et al., 2011). Furthermore, the DNA-damaging effect of *Glinus lotoides* L., fractions containing hopane-type saponins has been reported (Demma et al., 2013). Different classes of saponins compounds were identified in plant extracts of *Pterolobium stellatum* (Forssk.) Brenan., and proposed among the compounds responsible for the genotoxic effect. Some studies reported that the steroidal alkaloids from *Veratrum maackii* var. parviflorum (Maxim. ex Miq.) H. Hara., was genotoxic to the brain cells in mice (Cong et al., 2007). On the other hand, glycoalkaloids, solamargine and solasonine that were identified in *Solanum lycocarpum* A.St.-Hil. extracts were proposed to protect DNA damage.

Plant extracts contain a large variety of polyphenol compounds. The effects of polyphenols often in high doses in combination with known DNA-damaging agents have been studied in most animal experiments, and the results typically demonstrated protection (Azqueta and Collins, 2016). In addition, numerous cell culture tests have shown that high concentrations can cause DNA damage on their own; low concentrations, on the other hand, typically reduce DNA damage (Azqueta and Collins, 2016). Majority of the reviewed articles identified different phenolic and flavonoid including quercetin-3-O-glucopyranoside, chlorogenic acid, caffeic acid, and pnaphthoquinone that were proposed to be responsible of the genotoxic effect in *Rubus rosifoliu* Sm., *Eleutherine plicata (Mill.) Urb.*, *Artemisia vulgaris* L., and *Artemisia alba* Turra., extracts. Concerning the plant extracts with antigenotoxic effects of this reviewed article, several polyphenols were identified including chlorogenic and caffeic acids, apigenin 7-O-glucoside, rosmarinic acid, quercetin and apigenin rosmarinic acid were identified in *Dracocephalum moldavica* L., *Equisetum arvense* L., *Olea europaea* L*.,* leaves *Dracocephalum moldavica* L., and *Nigella satfia L.,* seed and oil. Hexadecanoic acid was identified and proposed to be the responsible phytochemicals for the DNA damage in *Bauhinia platypetala Burch. ex Benth.,* extract. In addition, hydrolase enzymes, like serine protease, were proposed as responsible genotoxic metabolites in *Sapindus saponaria* L., seeds extract. The active compound in *Phyllanthus emblica* L., fruit extract, geraniin, was suggested as the responsible phytochemical for the selective effect on chromosomal stability and instability in both cancerous and normal cells.

### 4.5 Mechanisms by which plant extracts exert their genotoxic effects

**FIGURE 8:**
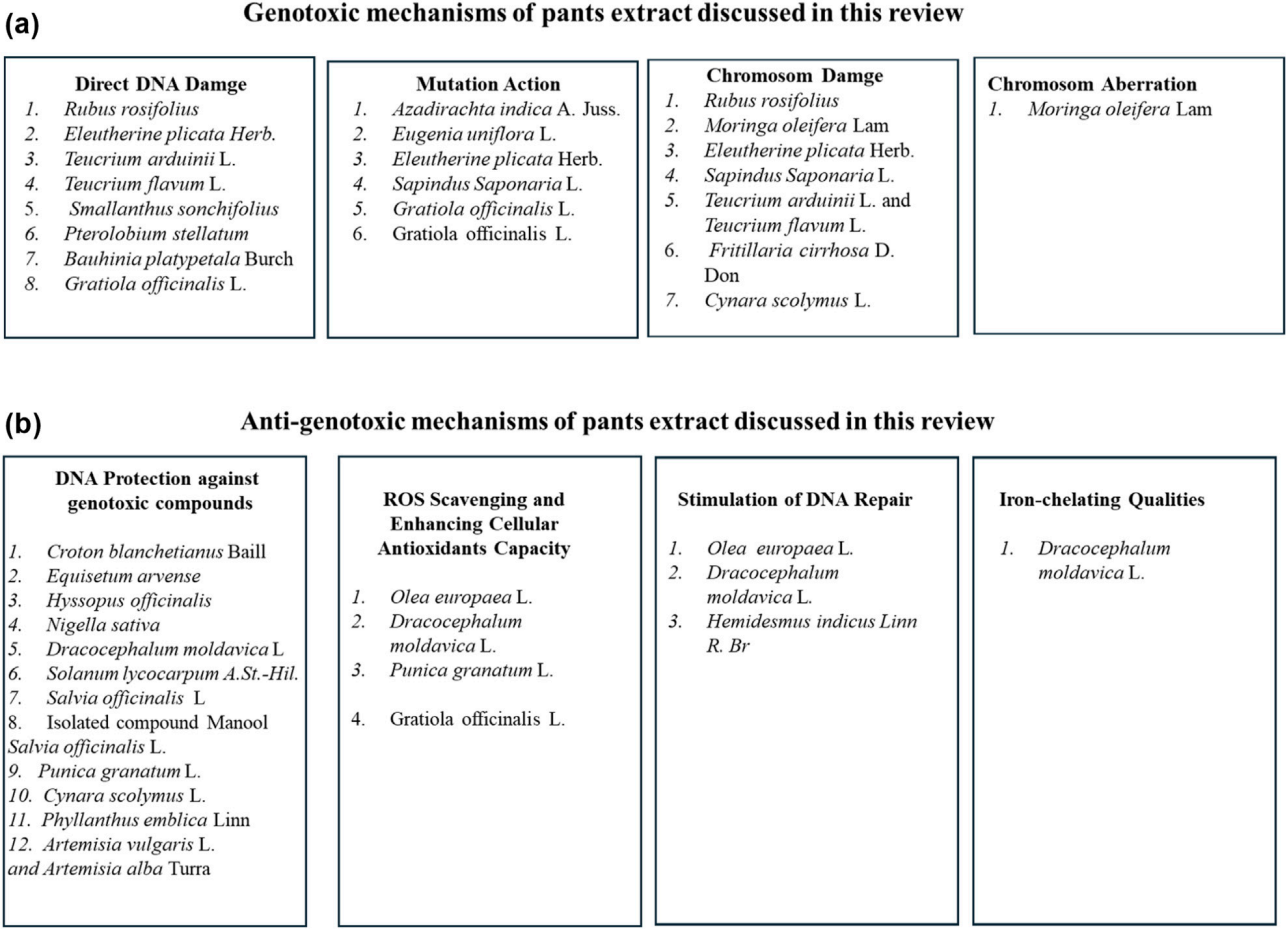
A summary of the genotoxic **(A)** and antigenotoxic **(B)** mechanisms of plant extracts discussed in this review.

Genotoxic substances cause damage to the genetic material within the cells by interacting with the structure and sequence of DNA. Few studies in the reviewed articles have shown how the plant extracts exerted genotoxic or antigenotoxic effects. For example, Quadros Gomes et al. (2021) proposed that oxidative stress, induction of apoptosis, and DNA alkylation are the mechanisms behind the genotoxic effect of *Eleutherine plicata* (Mill.) Urb., extract. Testing the genotoxicity of plant extracts poses specific challenges due to the complexity of the mixtures, which typically contain numerous constituents. The genotoxicity findings were often attributed to the synergistic effect of the plant extract mixture, including various classes of polyphenols, flavonoids, terpenes, saponins, and other compounds. Jacociunas et al. (2013) proposed that the genotoxic effect of the aqueous extract of *Cynara scolymus* L., leaves extract is due to the oxidation of certain compounds, leading to the generation of electrophilic compounds that can alkylate DNA. Most reviewed articles identified direct DNA damage as the mechanism of genotoxic plant extracts, as indicated by comet assay or chromosome damage as indicated by MN assay and chromosome aberration assay. Some plant extracts were found to have mutation effects. A summary of the genotoxic mechanisms of plant extracts discussed in this review is presented in Figure 8A.

Regarding antigenotoxic findings, most studies indicated that some plant extracts protected DNA against genotoxic compounds. Additionally, some plant extracts were reported to exhibit antigenotoxic mechanisms such as ROS scavenging and enhancing cellular antioxidant capacity. Stimulation of DNA repair and iron-chelating qualities were also among the antigenotoxic mechanisms reported by some plant extracts. A summary of the antigenotoxic mechanisms of plant extracts discussed in this review is shown in Figure 8B.

### 4.6 Future recommendations

*In vitro* studies have mostly been used to assess the genotoxicity of the plant extracts under consideration. *In vitro* investigations play an important role in initial screening; however, additional *in vivo* research utilizing appropriate models and assays should be conducted to confirm the findings. Based on the reviewed articles, focusing on plant extracts that have been studied for their antigenotoxicity potential *in vitro* and *in vivo*, with the identification of phytochemical compounds, it is critical to further test their active compounds and identify possible mechanisms of action for further application development. For instance, *Nigella sativa* L., seed oil and its active compounds. Furthermore, secoiridoid oleuropein and chlorogenic acid components have been suggested as the main compounds for the antigenotoxic effect of aqueous *Olea europea* L., leaf extracts. In addition, glycoalkaloids, solamargine, and solasonine phytochemicals were suggested to be responsible for the antigenotoxic effect of aqueous leaf extracts of *Solanum lycocarpum.*

## 5 Conclusion

Plant extracts used as medicine are perceived by the public as relatively safe, but, nowadays, the knowledge of the potential risks associated with these plant extracts increases. In utilizing pharmacologically active plant extracts, both beneficial and potential adverse effects must be considered. Evaluation of the genotoxicity potential of plant extracts that are used as traditional medicine is essential to ensure that they are safe for use and in the search for new medication. In this narrative review, genotoxicity studies of 28 plant extracts used as a medicine with several reported biological activities were discussed. We focused mainly on the plant extracts with identified phytochemicals that were proposed to be responsible for the genotoxicity effect under experimental conditions. Most of the reviewed articles proposed that the synergistic effect of different phytochemicals compounds present in plant extracts contributed to their genotoxicity response. Moreover, most of the studies dialed with crude extracts which contain a mixture of different phytochemicals, and the specific bioactive molecules responsible for such effects, as well as the basic mechanisms by which they interact with DNA and cause damage or protect DNA damage, remain unidentified for most of the plant extracts reviewed. Hence, finding and understanding the genotoxic or genoprotactive effect of the major active compounds separately is critical for further study and development of employing these plant extracts in medicine. Based on the information gathered from the studies reviewed, it is difficult to mention about the sensitivity, false positive, or false negative of the methods employed to evaluate the genotoxicity of selected plant extracts. Among the studies reviewed, 14 studies demonstrated positive genotoxic outcomes. However, out of these, 8 studies assessed genotoxicity through one assay *in vitro* methods. Here, we recommend the necessity of incorporating genotoxic assays in both bacterial and mammalian species, ensuring at least one *in vivo* test is included, and following the same administration route as suggested for medicinal use thus addressing all potential forms of DNA damage and ensuring improved safety for the community. It is difficult to draw a conclusion from this review about the genotoxicity response of selected plant extracts because the findings were based on the experimental conditions that depend on many aspects including different assays used, different plant parts, extraction methods and solvents, concentrations, doses, and plant location. Finally, the study of the genotoxicity of medicinal plants is extremely important since they are widely used in traditional medicine and as a raw material for the development of new herbal medicines or synthesis medicine. Therefore, more research on the genotoxicity of medicinal plant extract is required.

## References

[B1] Abdel-Moneim A. M. Essawy A. E. Hamed S. S. Abou-Gabal A. A. Alzergy A. A. (2017). Protective effect of *Nigella sativa* seeds against spermatocyte chromosomal aberrations and genotoxicity induced by carbon tetrachloride in mice. Environ. Sci. Pollut. Res. Int. 24, 11677–11682. 10.1007/s11356-017-8806-y 28324259

[B2] Abdul Q. A. Seong S. H. Hui S. Ahn B. R. Islam M. N. Jung H. A. (2018). Anti-inflammatory potential of Artemisia capillaris and its constituents in LPS-induced RAW264.7 cells. Nat. Prod. Sci. 24, 171–218. 10.20307/nps.2018.24.3.171

[B3] Akaneme F. Amaefule C. C. (2012). Evaluation of the cytotoxicity and genotoxicity of aqueous leaf extracts of Azadirachta indica A. Juss using the Allium test. JMPR 6, 898–3907. 10.5897/jmpr12.427

[B5] Al-Anizi A. A. Hellyer M. T. Zhang D. (2014). Toxicity assessment and modelling of *Moringa oleifera* seeds in water purification by whole cell bioreporter. Water Rese 56, 77–87. 10.1016/j.watres.2014.02.045 24657325

[B6] Al-Naqeep G. Al-Zubairi A. S. Ismail M. Amom Z. H. Esa N. M. (2011). Antiatherogenic potential of *Nigella sativa* seeds and oil in diet-induced hypercholesterolemia in rabbits. Evid. Based Complement. Altern. Med. 2011, e213628. 10.1093/ecam/neq071 PMC3136238 21792359

[B7] Al-Okbi S. Y. Mohamed D. A. Hamed T. E. Edris A. E. Fouda K. (2018). Hepatic regeneration and reno-protection by fish oil, *Nigella sativa* oil and combined fish oil/*Nigella sativa* volatiles in CCl4 treated rats. J. Oleo Sci. 67, 345–353. 10.5650/jos.ess17204 29459508

[B8] Alves A. B. dos Santos R. S. Calil S. Niero R. Lopes J. Perazzo F. F. (2014). Genotoxic assessment of *Rubus imperialis* (Rosaceae) extract *in vivo* and its potential chemoprevention against cyclophosphamide-induced DNA damage. J. Ethnopharmacol. 153, 694–700. 10.1016/j.jep.2014.03.033 24685582

[B9] Ananthi R. Chandra N. Santhiya S. T. Ramesh A. (2010). Genotoxic and antigenotoxic effects of Hemidesmus indicus R. Br. root extract in cultured lymphocytes. J. Ethnopharmacol. 127, 558–560. 10.1016/j.jep.2009.10.034 19896526

[B10] Andrade A. F. Alves J. M. Corrêa M. B. Cunha W. R. Veneziani R. C. S. Tavares D. C. (2016). *In vitro* cytotoxicity, genotoxicity and antigenotoxicity assessment of Solanum lycocarpum hydroalcoholic extract. Pharm. Biol. 2, 2786–2790. 10.1080/13880209.2016.1175022 27159582

[B11] Andualem G. Umar S. Getnet F. Tekewe A. Alemayehu H. Kebede N. (2014). Antimicrobial and phytochemical screening of methanol extracts of three medicinal plants in Ethiopia. Advan. Biol. Res. 8, 101–106. 10.5829/idosi.abr.2014.8.3.8383

[B12] Anto E. J. Syahputra R. A. Silitonga H. A. Situmorang P. C. Nugaraha S. E. (2022). Oral acute toxicity study extract ethanol of balakka fruit (Phyllanthus emblica). Pharmacia 69 (1), 187–194. 10.3897/pharmacia.69.e81280

[B13] Aprotosoaie A. C. Mihai C. T. Vochița G. Rotinberg P. Trifan A. Luca S. V. (2016). Antigenotoxic and antioxidant activities of a polyphenolic extract from *European Dracocephalum moldavica* L. Ind. Crops Prod. 79, 248–257. 10.1016/j.indcrop.2015.11.004

[B14] Araújo S. Fernandes T. C. Cardona Y. T. Almeida P. M. Marin-Morales M. A. Dos Santos A. V. (2015). Cytotoxic and genotoxic effects of ethanolic extract of *Euphorbia hyssopifolia* L. on HepG2 cells. J. Ethnopharmacol. 170, 16–19. 10.1016/j.jep.2015.04.044 25937254

[B15] Arslan I. Ili P. (2015). Genotoxicological assessment of nebuloside-A a triterpenoid saponin compound on whole blood DNA. Int. J. Food Prop. 18, 2374–2379. 10.1080/10942912.2014.971185

[B16] Asare G. A. Gyan B. Bugyei K. Adjei S. Mahama R. Addo P. (2012). Toxicity potentials of the nutraceutical *Moringa oleifera* at supra-supplementation levels. J. Ethnopharmacol. 139, 265–272. 10.1016/j.jep.2011.11.009 22101359

[B17] Ayerza R. (2011). Seed yield components, oil content, and fatty acid composition of two cultivars of moringa (Moringa oleifera Lam.) growing in the Arid Chaco of Argentina. Ind. CROP Prod. 33, 389–394. 10.1016/j.indcrop.2010.11.003

[B18] Aziz K. Nowsheen S. Pantelias G. Iliakis G. Gorgoulis V. G. Georgakilas A. G. (2012). Targeting DNA damage and repair: embracing the pharmacological era for successful cancer therapy. Pharmacol. Ther. 133, 334–350. 10.1016/j.pharmthera.2011.11.010 22197993

[B19] Azqueta A. Collins A. (2016). Polyphenols and DNA damage: a mixed blessing. Nutrients 8, 785. 10.3390/nu8120785 27918471 PMC5188440

[B20] Azzini E. Bugianesi R. Romano F. Di Venere D. Miccadei S. Durazzo A. (2007). Absorption and metabolism of bioactive molecules after oral consumption of cooked edible heads of Cynara scolymus L. (cultivar Violetto di Provenza) in human subjects: a pilot study. Br. J. Nutr. 97, 963–969. 10.1017/S0007114507617218 17408528

[B21] Bakare A. A. Akpofure A. Gbadebo A. M. Fagbenro O. S. Oyeyemi I. T. (2022). Aqueous extract of *Moringa oleifera* Lam. induced mitodepression and chromosomal aberration in Allium cepa, and reproductive genotoxicity in male mice. Adv. Tradit. Med. 22, 685–695. 10.1007/s13596-021-00564-9

[B23] Baldissera M. D. Souza C. F. Dolci G. S. Grando T. H. Sagrillo M. Vaucher R. A. (2017). Monoterpene alpha-terpinene induced hepatic oxidative, cytotoxic and genotoxic damage is associated to caspase activation in rats. J. Appl. Biomed. 15, 187–195. 10.1016/j.jab.2017.01.002

[B24] Balunas M. J. Kinghorn A. D. (2005). Drug discovery from medicinal plants. Life Sci. 78, 431–441. 10.1016/j.lfs.2005.09.012 16198377

[B25] Barcellona C. S. Cabrera W. M. Honoré S. M. Mercado M. I. Sánchez S. S. Genta S. B. (2012). Safety assessment of aqueous extract from leaf *Smallanthus sonchifolius* and its main active lactone, enhydrin. J. Ethnopharmacol. 144, 362–370. 10.1016/j.jep.2012.09.021 23000115

[B26] Bardoloi A. Soren A. D. (2022). Genotoxicity induced by medicinal plants. Bull. Natl. Res. Cent. 46, e119. 10.1186/s42269-022-00803-2

[B27] Baričevič D. Bartol T. (2000). “The biological/pharmacological activity of the salvia genus,” in The genus Salvia. Editor Kintzios S. E. (Amsterdam: Harwood Academic Publishers), 143–184.

[B28] Beeharry N. Chambers J. A. Green I. C. (2004). Fatty acid protection from palmitic acid-induced apoptosis is lost following PI3-kinase inhibition. Apoptosis 9, 599–607. 10.1023/B:APPT.0000038039.82506.0c 15314288

[B29] Bellakhdar J. Claisse R. Fleurentin J. Younos C. (1991). Repertory of standard herbal drugs in the Moroccan pharmacopoea. J. Ethnopharmacol. 35, 123–143. 10.1016/0378-8741(91)90064-k 1809818

[B30] Bellomaria B. Arnold N. Valentini G. (1998). Essential Oil of *Teucrium flavum subsp. hellenicum* from Greece. J. Essent. Oil Res. 10, 131–133. 10.1080/10412905.1998.9700863

[B31] Bhattacharya S. (2011). Natural antimutagens: a review. Res. J. Med. Plant 5, 116–126. 10.3923/rjmp.2011.116.126

[B32] Bhavsar S. Chandel D. (2022). Cytotoxic and genotoxic effects *of Kalanchoe pinnata* (Lam.) Pers. fresh leaf juice in the cultured human blood lymphocytes. Drug Chem. Toxicol. 45, 360–366. 10.1080/01480545.2019.1696814 31791148

[B33] Bocayuva Tavares G. D. Fortes Aiub C. A. Felzenszwalb I. Carrão Dantas E. K. Araújo-Lima C. F. Siqueira Júnior C. L. (2021). *In vitro* biochemical characterization and genotoxicity assessment of *Sapindus saponaria* seed extract. J. Ethnopharmacol. 276, 114170. 10.1016/j.jep.2021.114170 33932515

[B202] Bouguellid G. Russo C. Lavorgna M. Piscitelli C. Ayouni K. Wilson E. (2020). Antimutagenic, antigenotoxic and antiproliferative activities of *Fraxinus angustifolia* Vahl. leaves and stem bark extracts and their phytochemical composition. PLoS One 15 (4), e0230690. 10.1371/journal.pone.0230690 32298276 PMC7161964

[B34] Čabarkapa A. Dekanski D. Živković L. Milanović-Čabarkapa M. Bajić V. Topalović D. (2017). Unexpected effect of dry olive leaf extract on the level of DNA damage in lymphocytes of lead intoxicated workers, before and after CaNa_2_EDTA chelation therapy. Food Chem. Toxicol. 106, 616–623. 10.1016/j.fct.2016.12.023 28011361

[B35] Cabarkapa A. Zivković L. Zukovec D. Djelić N. Bajić V. Dekanski D. (2014). Protective effect of dry olive leaf extract in adrenaline induced DNA damage evaluated using *in vitro* comet assay with human peripheral leukocytes. Toxicol Vitro 28, 451–456. 10.1016/j.tiv.2013.12.014 24389114

[B36] Castro A. Cruz J. N. Sodré D. F. Correa-Barbosa J. Azonsivo R. de Oliveira M. S. (2021). Evaluation of the genotoxicity and mutagenicity of isoeleutherin and eleutherin isolated from Eleutherine plicata herb. using bioassays and *in silico* approaches. Arab. J. Chem. 14, 103084. 10.1016/j.arabjc.2021.103084

[B37] Cetojević-Simin D. D. Canadanović-Brunet J. M. Bogdanović G. M. Djilas S. M. Cetković G. S. Tumbas V. T. (2010). Antioxidative and antiproliferative activities of different horsetail (*Equisetum arvense* L.) extracts. J. Med. Food 13, 452–459. 10.1089/jmf.2008.0159 20170379

[B39] Chaowuttikul C. Palanuvej C. Ruangrungsi N. (2020). Quantification of chlorogenic acid, rosmarinic acid, and caffeic acid contents in selected Thai medicinal plants using RP-HPLC-DAD. Braz. J. Pharm. Sci. 56, e17547. 10.1590/s2175-97902019000317547

[B209] Cong Y. Guo L. Yang J. Y. Li L. Zhou Y. B. Chen J. (2007). Steroidal alkaloids from *Veratrum japonicum* with genotoxicity on brain cell DNA of the cerebellum and cerebral cortex in mice. Planta Med. 73 (15), 1588–1591. 10.1055/s-2007-993750 18074316

[B40] Couto C. L. Moraes D. F. Agenes M. D. Amaral F. M. Guerra R. N. (2016). *Eleutherine bulbous* (Mill.) Urb.: a review study. J. Med. Plants Res. 10, 286–297. 10.5897/JMPR2016.6106

[B41] da Cunha F. A. B. Waczuk E. P. Duarte A. E. Barros L. M. Elekofehinti O. O. Matias E. F. F. (2016). Cytotoxic and antioxidative potentials of ethanolic extract of Eugenia uniflora L. (Myrtaceae) leaves on human blood cells. Biomed. Pharmacother. 84, 614–621. 10.1016/j.biopha.2016.09.089 27694006

[B42] Dall’acqua S. Cervellati R. Loi M. C. Innocenti G. (2008). Evaluation of *in vitro* antioxidant properties of some traditional Sardinian medicinal plants: investigation of the high antioxidant capacity of *Rubus ulmifolius* . Food Chem. 106, 745–749. 10.1016/j.foodchem.2007.06.055

[B43] Dall’Agnol R. Lino von Poser G. L. (2000). The use of complex polysaccharides in the management of metabolic diseases: the case of Solanum lycocarpum fruits. J. Ethnopharmacolo 71, 337–341. 10.1016/s0378-8741(00)00165-3 10904183

[B44] Dantas F. G. D. S. Castilho P. F. Almeida-Apolonio A. A. Araújo R. P. Oliveira K. M. P. (2020). Mutagenic potential of medicinal plants evaluated by the Ames Salmonella/microsome assay: a systematic review. Mutat. Res. Rev. Mutat. Res. 786, 108338. 10.1016/j.mrrev.2020.108338 33339578

[B46] da Silva R. P. Jacociunas L. V. de Carli R. F. de Abreu B. R. R. Lehmann M. da Silva J. (2017). Genotoxic and chemopreventive assessment of Cynara scolymus L. aqueous extract in a human-derived liver cell line. Drug Chem. Toxicol. 40, 484–488. 10.1080/01480545.2017.1279625 28147701

[B47] Dassprakash M. V. Arun R. Abraham S. K. Premkumar K. (2012). *In vitro* and *in vivo* evaluation of antioxidant and antigenotoxic potential of Punica granatum leaf extract. Pharm. Biol. 50, 1523–1530. 10.3109/13880209.2012.689771 22963679

[B48] Dastmalchi K. Dorman D. H. J. Laakso I. Hiltunen R. (2007). Chemical composition and antioxidative activity of Moldavian balm (*Dracocephalum moldavica* L.) extracts. LWT - Food Sci. Technol. 40, 1655–1663. 10.1016/j.lwt.2006.11.013

[B49] de Barros M. C. Silva A. G. B. Souza T. G. D. S. Chagas C. A. Machado J. C. B. Ferreira M. R. A. (2022). Evaluation of acute toxicity, 28-day repeated dose toxicity, and genotoxicity of *Moringa oleifera* leaves infusion and powder. J. Ethnopharmacol. 296, e115504. 10.1016/j.jep.2022.115504 35760258

[B51] de Melo Candeia G. L. O. Costa W. K. de Oliveira A. M. Napole˜ ao T. H. Guedes Paiva P. M. Ferreira M. R. A. (2022). Anti-inflammatory, antinociceptive effects and involvement of opioid receptors in the antinociceptive activity of *Eugenia uniflora* leaves obtained with water, ethanol, and propylene glycol mixture. J. Ethnopharmacol. 296, e115508. 10.1016/j.jep.2022.115508 35779820

[B52] Demma J. El-Seedi H. Engidawork E. Aboye T. L. Göransson U. Hellman B. (2013). An *in vitro* study on the DNA damaging effects of phytochemicals partially isolated from an extract of *Glinus lotoides* . Phytother. Res. 27, 507–514. 10.1002/ptr.4744 22648529

[B53] de Oliveira A. M. de Freitas A. F. Costa W. K. Machado J. C. Bezerra I. C. Ferreira M. R. (2022b). Flavonoid-rich fraction of *Croton blanchetianus* Baill. (Euphorbiaceae) leaves: chemical profile, acute and subacute toxicities, genotoxicity, and antioxidant potential. S. Afr. J. Bot. 144, 238–249. 10.1016/j.sajb.2021.08.040

[B54] de Oliveira A. M. Widmer R. do Nascimento M. F. Costa W. K. Paiva P. M. G. Napoleão T. H. (2022a). Flavonoid-rich fraction from *Croton blanchetianus* (Euphorbiaceae) leaves exerts peripheral and central analgesic effects by acting via the opioid and cholinergic systems. Chem. Biodivers. 19, e202100853. 10.1002/cbdv.202100853 34990049

[B55] De Oliveira N. C. Sarmento M. S. Nunes E. A. Porto C. M. Rosa D. P. Bona S. R. (2012). Rosmarinic acid as a protective agent against genotoxicity of ethanol in mice. Food Chem. Toxicol. 50, 1208–1214. 10.1016/j.fct.2012.01.028 22306517

[B56] De Quadros A. P. O. Oshiiwa B. Petreanu M. Niero R. Rosa P. C. P. Sawaya A. C. H. F. (2023). *Rubus rosifolius* (Rosaceae) stem extract induces cell injury and apoptosis in human hepatoma cell line. Toxicol Vitro 86, e105485. 10.1016/j.tiv.2022.105485 36279965

[B57] de Souza P. Boeing T. Somensi L. B. Cechinel-Zanchett C. C. Bastos J. K. Petreanu M. (2017). Diuretic effect of extracts, fractions and two compounds 2α,3β,19α-trihydroxy-urs-12-en-28-oic acid and 5-hydroxy-3,6,7,8,4'-pentamethoxyflavone from *Rubus rosaefolius* Sm. (Rosaceae) leaves in rats. Naunyn Schmiedeb. Arch. Pharmacol. 390, 351–360. 10.1007/s00210-016-1333-4 28013356

[B58] Diab K. A. Fahmy M. A. Hassan Z. M. Hassan E. M. Salama A. B. Omara E. A. (2018). Genotoxicity of carbon tetrachloride and the protective role of essential oil *of Salvia officinalis* L. in mice using chromosomal aberration, micronuclei formation, and comet assay. Environ. Sci. Pollut. Res. Int. 25, 1621–1636. 10.1007/s11356-017-0601-2 29098592

[B59] Dormousoglou M. Efthimiou I. Antonopoulou M. Fetzer D. L. Hamerski F. Corazza M. L. (2022). Investigation of the genotoxic, antigenotoxic and antioxidant profile of different extracts from *Equisetum arvense* L. Antioxidants 11, 1393. 10.3390/antiox11071393 35883882 PMC9312020

[B60] Durnova N. A. Kurchatova M. N. (2015). The effect of plant extracts on the cyclophosphamide induction of micronucleus in red blood cells of outbred white mice. Cell Tissue Biol. 5, 452–458. 26495712

[B61] Džamic A. Sokovic M. Novakovic M. Jadranin M. Ristic M. Teševic V. (2013). Composition, antifungal and antioxidant properties of *Hyssopus officinalis* L. subsp. pilifer (Pant.) Murb. essential oil and deodorized extracts. Ind. Crops Prod. 51, 401–407. 10.1016/j.indcrop.2013.09.038

[B62] Efeoglu E. Maher M. A. Casey A. Byrne H. J. (2018). Toxicological assessment of nanomaterials: the role of *in vitro* Raman microspectroscopic analysis. Anal. Bioanal. Chem. 410, 1631–1646. 10.1007/s00216-017-0812-x 29264675

[B63] Elespuru R. K. Doak S. H. Collins A. R. Dusinska M. Pfuhler S. Manjanatha M. (2022). Common considerations for genotoxicity assessment of nanomaterials. Front. Toxicol. 4, 859122. 10.3389/ftox.2022.859122 35686044 PMC9171035

[B64] Ellinger-Ziegelbauer H. Aubrecht J. Kleinjans J. C. Ahr H. J. (2009). Application of toxicogenomics to study mechanisms of genotoxicity and carcinogenicity. Toxicol. Lett. 186, 36–44. 10.1016/j.toxlet.2008.08.017 18822359

[B65] Engen A. Maeda J. Wozniak D. E. Brents C. A. Bell J. J. Uesaka M. (2015). Induction of cytotoxic and genotoxic responses by natural and novel quercetin glycosides. Mutat. Res. Genet. Toxicol. Environ. Mutagen 784-785, 15–22. 10.1016/j.mrgentox.2015.04.007 26046972

[B66] Enke C. G. Nagels L. J. (2011). Undetected components in natural mixtures: how many? What concentrations? Do they account for chemical noise? What is needed to detect them? Anal. Chem. 83, 2539–2546. 10.1021/ac102818a 21366323

[B67] European Food Safety Authority (2011). Scientific Opinion on genotoxicity testing strategies applicable to food and feed safety assessment. EFSA J. 9, 2379. 10.2903/j.efsa.2011.2379

[B203] Falcão T. R. de Araújo A. A. Soares L. A. L. de Moraes Ramos R. T. Bezerra I. C. F. Ferreira M. R. A. (2018). Crude extract and fractions from *Eugenia uniflora* Linn leaves showed anti-inflammatory, antioxidant, and antibacterial activities MC Complement Altern. Med. 2018. 10.1186/s12906-018-2144-6 PMC5845158 29523111

[B68] Felício L. P. Silva E. M. Ribeiro V. Miranda C. T. Vieira I. L. Passos D. C. (2011). Mutagenic potential and modulatory effects of the medicinal plant Luehea divaricata (Malvaceae) in somatic cells of *Drosophila melanogaster*: SMART/wing. Genet. Mol. Res. 10, 16–24. 10.4238/vol10-1gmr982 21218382

[B69] Ferreira M. R. A. Daniele-Silva A. Ferreira de Almeida L. dos Santos E. C. F. Machado J. C. B. de Oliveira A. M. (2022). Safety evaluation of aqueous extract from *Eugenia uniflora* leaves: acute and subacute toxicity and genotoxicity *in vivo* assays. J. Ethnopharmacol. 298, e115668. 10.1016/j.jep.2022.115668 36038093

[B70] Franco-Ramos R. S. López-Romero C. A. Torres-Ortega H. Oseguera-Herrera D. Lamoreaux-Aguayo J. P. Molina-Noyola D. (2020). Evaluation of anti-cytotoxic and anti-genotoxic effects of *Nigella sativa* through a micronucleus test in BALB/c mice. Nutrients 12, 1317. 10.3390/nu12051317 32384595 PMC7284975

[B71] Frank F. M. Ulloa J. Cazorla S. I. Maravilla G. Malchiodi E. L. Grau A. (2013). Trypanocidal activity of Smallanthus sonchifolius: identification of active sesquiterpene lactones by bioassay-guided fractionation. Evid. Based Complement. Altern. Med. 2013, e627898. 10.1155/2013/627898 PMC3690263 23840260

[B72] Fratianni F. Tucci M. De Palma M. Pepe R. Nazzaro F. (2007). Polyphenolic composition in different parts of some cultivars of globe artichoke (Cynara cardunculus L. var. scolymus (L.) Fiori). Food Chem. 104, 1282–1286. 10.1016/j.foodchem.2007.01.044

[B73] Freitas A. F. S. Costa W. K. Machado J. C. B. Ferreira M. R. A. Paiva P. M. G. Medeiros P. L. (2020). Toxicity assessment and antinociceptive activity of an ethanolic extract from Croton blanchetianus (Euphorbiaceae) leaves. S Afr. J. Bot. 133, 30–39. 10.1016/j.sajb.2020.06.015

[B74] Fürer K. Simões-Wüst A. P. von Mandach U. Hamburger M. Potterat O. (2016). Bryophyllum pinnatum and related species used in anthroposophic medicine: constituents, pharmacological activities, and clinical efficacy. Planta Medica 82, 930–941. 10.1055/s-0042-106727 27220081

[B75] Galhena B. P. Samarakoon S. S. R. Thabrew M. I. Paul S. F. D. Perumal V. Mani C. (2017). Protective effect of a polyherbal aqueous extract comprised of *Nigella sativa* (seeds), Hemidesmus indicus (roots), and Smilax glabra (rhizome) on bleomycin induced cytogenetic damage in human lymphocytes. Biomed. Res. Int. 2017, e1856713. 10.1155/2017/1856713 PMC5463188 28626752

[B76] Garcia D. Ramos A. J. Sanchis V. Marín S. (2012). Effect of *Equisetum arvense* and *Stevia rebaudiana* extracts on growth and mycotoxin production by Aspergillus flavus and Fusarium verticillioides in maize seeds as affected by water activity. Int. J. Food Microbiol. 153, 21–27. 10.1016/j.ijfoodmicro.2011.10.010 22104120

[B77] Genta S. B. Cabrera W. M. Mercado M. I. Grau A. Catalán C. A. Sánchez S. S. (2010). Hypoglycemic activity of leaf organic extracts from *Smallanthus sonchifolius*: constituents of the most active fractions. Chem. Biol. Interact. 185, 143–152. 10.1016/j.cbi.2010.03.004 20211156

[B78] George S. Tushar K. V. Unnikrishnan K. P. Hashim K. M. Balachandran I. (2008). *Hemidesmus indicus* (L.) R. Br. A review. J. Plant Sci. 3, 146–156. 10.3923/jps.2008.146.156

[B79] Gil-Izquierdo A. Gil M. I. Conesa M. A. Ferreres F. (2001). The effect of storage temperatures on vitamin C and phenolics content of artichoke (Cynara scolymus L.) heads. Innov. Food Sci. Emerg. Technol. 2, 199–202. 10.1016/S1466-8564(01)00018-2

[B80] Goldsmith C. D. Vuong Q. V. Sadeqzadeh E. Stathopoulos C. E. Roach P. D. Scarlett C. J. (2015). Phytochemical properties and anti-proliferative activity of *Olea europaea* L. leaf extracts against pancreatic cancer cells. Molecules 20, 12992–13004. 10.3390/molecules200712992 26193251 PMC6332116

[B81] Gordanian B. Behbahani M. Carapetian J. Fazilati M. (2014). *In vitro* evaluation of cytotoxic activity of flower, leaf, stem and root extracts of five Artemisia species. Res. Pharm. Sci. 9, 91–96. 25657777 PMC4311295

[B82] Grujičić D. Marković A. Vukajlović J. T. Stanković M. Jakovljević M. R. Ćirić A. (2020). Genotoxic and cytotoxic properties of two medical plants (*Teucrium arduini* L. and *Teucrium flavum* L.) in relation to their polyphenolic contents. Mutat. Res. Genet. Toxicol. Environ. Mutagen 852, e503168. 10.1016/j.mrgentox.2020.503168 32265044

[B83] Guo X. Ni J. Liu X. Xue J. Wang X. (2013). Phyllanthus emblica L. fruit extract induces chromosomal instability and suppresses necrosis in human colon cancer cells. Int. J. Vitam. Nutr. Res. 83, 271–280. 10.1024/0300-9831/a000169 25305222

[B84] Guo X. Ni J. Xue J. Wang X. (2017a). Extract of bulbus *Fritillaria cirrhosa* perturbs spindle assembly checkpoint, induces mitotic aberrations and genomic instability in human colon epithelial cell line. Exp. Toxicol. Pathol. 69, 163–171. 10.1016/j.etp.2016.12.009 28073664

[B85] Guo X. Wang C. Tian W. Dai X. Ni J. Wu X. (2021). Extract of bulbus of *Fritillaria cirrhosa* induces spindle multipolarity in human-derived colonic epithelial NCM460 cells through promoting centrosome fragmentation. Mutagenesis 36, 95–107. 10.1093/mutage/geab002 33450026

[B86] Guo X. Wang H. Ni J. Liang Z. Wu X. Xue J. (2018). Geraniin selectively promotes cytostasis and apoptosis in human colorectal cancer cells by inducing catastrophic chromosomal instability. Mutagenesis 33 (4), 271–281. 10.1093/mutage/gey016 30085224

[B87] Guo X. Wang X. (2016). Phyllanthus emblica fruit extract activates spindle assembly checkpoint, prevents mitotic aberrations and genomic instability in human colon epithelial NCM460 cells. Int. J. Mol. Sci. 17, 1437. 10.3390/ijms17091437 27598149 PMC5037716

[B88] Guo X. Wu X. Ni J. Zhang L. Xue J. Wang X. (2020). Aqueous extract of bulbus Fritillaria cirrhosa induces cytokinesis failure by blocking furrow ingression in human colon epithelial NCM460 cells. Mutat. Res. Genet. Toxicol. Environ. Mutagen 850-851, e503147. 10.1016/j.mrgentox.2020.503147 32247562

[B89] Guo X. H. Ni J. Xue J. L. Wang X. (2017b). Phyllanthus emblica Linn. fruit extract potentiates the anticancer efficacy of mitomycin C and cisplatin and reduces their genotoxicity to normal cells *in vitro* . J. Zhejiang Univ. Sci. B 18 (12), 1031–1045. 10.1631/jzus.B1600542 29204983 PMC5742286

[B90] Gupta S. Jain R. Kachhwaha S. Kothari S. L. (2017). Nutritional and medicinal applications of *Moringa oleifera* Lam. Review of current status and future possibilities. J. Herb. Med. 11, 1–11. 10.1016/j.hermed.2017.07.003

[B91] Hamilton A. C. (2004). Medicinal plants, conservation and livelihoods. Biodivers. Conserv. 13, 1477–1517. 10.1023/B:BIOC.0000021333.23413.42

[B92] Hao D. C. Gu X. J. Xiao P. G. Peng Y. (2013). Phytochemical and biological research of Fritillaria medicine resources. Chin. J. Nat. Med. 11, 330–344. 10.1016/S1875-5364(13)60050-3 23845541

[B93] Ho C. T. Wang M. Wei G. J. Huang T. C. Huang M. T. (2000). Chemistry and antioxidative factors in rosemary and sage. BioFactors 13, 161–166. 10.1002/biof.5520130126 11237177

[B94] Islam M. T. (2017). A review on genotoxic and mutagenic effects of monoterpenes. Int. J. Med. 5, 220–222. 10.14419/ijm.v5i2.8035

[B95] Jacociunas L. V. de Andrade H. H. Lehmann M. de Abreu B. R. Ferraz A. deB. da Silva J. (2013). Artichoke induces genetic toxicity in the cytokinesis-block micronucleus (CBMN) cytome assay. Food Chem. Toxicol. 55, 56–59. 10.1016/j.fct.2012.12.024 23274746

[B96] Jaijoy K. Soonthornchareonnon N. Lertprasertsuke N. Panthong A. Sireeratawong S. (2010). Acute and chronic oral toxicity of standardized water extract from the fruit of Phyllanthus emblica Linn. Int. J. J Appl Res Natl Prod 3, 48–58.

[B97] Jakovljević M. R. Grujičić D. Vukajlović J. T. Markovic A. Milutinović M. G. Stanković M. S. (2020). *In vitro* study of genotoxic and cytotoxic activities of methanol extracts of *Artemisia vulgaris* L. and *Artemisia alba Turra* . S Afr. J. Bot. Journa 132, 117–126. 10.1016/j.sajb.2020.04.016

[B98] Jiang J. Yuan X. Wang T. Chen H. Zhao H. Yan X. (2014). Antioxidative and cardioprotective effects of total flavonoids extracted from *Dracocephalum moldavica* L. against acute ischemia/reperfusion-induced myocardial injury in isolated rat heart. Cardiovasc Toxicol. 14, 174–182. 10.1007/s12012-013-9221-3 24395711

[B99] Jiang Q. W. Chen M. W. Cheng K. J. Yu P. Z. Wei X. Shi Z. (2016). Therapeutic potential of steroidal alkaloids in cancer and other diseases. Med. Res. Rev. 36, 119–143. 10.1002/med.21346 25820039

[B100] Junio H. A. Sy-Cordero A. A. Ettefagh K. A. Burns J. T. Micko K. T. Graf T. N. (2011). Synergy-directed fractionation of botanical medicines: a case study with goldenseal (Hydrastis canadensis). J. Nat. Prod. 74, 1621–1629. 10.1021/np200336g 21661731 PMC3142294

[B101] Jurenka J. S. (2008). Therapeutic applications of pomegranate (Punica granatum L.): a review. Altern. Med. Rev. 13, 128–144. 18590349

[B102] Kahaliw W. Hellman B. Engidawork E. (2018). Genotoxicity study of Ethiopian medicinal plant extracts on HepG2 cells. BMC Complement. Altern. Med. 18, 45. 10.1186/s12906-017-2056-x 29391002 PMC5796566

[B103] Kane N. F. Kyama M. C. Nganga J. K. Hassanali A. Diallo M. Kimani F. T. (2019). Comparison of phytochemical profiles and antimalarial activities of Artemisia afra plant collected from five countries in Africa. S Afr. J. Bot. Journa 125, 126–133. 10.1016/j.sajb.2019.07.001

[B104] Kane S. R. Adnaik R. S. Apte A. A. Magdum C. S. (2009). Laxative and anti-helmintic activity of aqueous extract of Euphorbia thymifolia Linn. RJP 1, 182–184.

[B105] Kanegusuku M. Sbors D. Bastos E. S. de Souza M. M. Cechinel-Filho V. Yunes R. A. (2007). Phytochemical and analgesic activity of extract, fractions and a 19-hydroxyursane-type triterpenoid obtained from *Rubus rosaefolius* (Rosaceae). Biol. Pharm. Bull. 30, 999–1002. 10.1248/bpb.30.999 17473451

[B106] Kang S. H. Kwon J. Y. Lee J. K. Seo Y. R. (2013). Recent advances in *in vivo* genotoxicity testing: prediction of carcinogenic potential using comet and micronucleus assay in animal models. J. Cancer Prev. 18, 277–288. 10.15430/jcp.2013.18.4.277 25337557 PMC4189446

[B107] Keesing F. Ostfeld R. S. (2021). Impacts of biodiversity and biodiversity loss on zoonotic diseases. Perspective 118, e2023540118. 10.1073/pnas.2023540118 PMC8092607 33820825

[B108] Khor K. Z. Lim V. Moses E. J. Abdul Samad N. (2018). The *in vitro* and *in vivo* anticancer properties of moringa oleifera. Evid. Based Complement. Altern. Med. 2018, e1071243. 10.1155/2018/1071243 PMC6261394 30538753

[B109] Kigen G. Maritim A. Some F. Kibosia J. Rono H. Chepkwony S. (2016). Ethnopharmacological survey of the medicinal plants used in tindiret, nandi county, Kenya. Afr. J. Tradit. Complement. Altern. Med. 13, 156–168. 10.4314/ajtcam.v13i3.19

[B110] Kirkland D. Aardema M. Henderson L. Müller L. (2005). Evaluation of the ability of a battery of three *in vitro* genotoxicity tests to discriminate rodent carcinogens and non-carcinogens I. Sensitivity, specificity and relative predictivity. Mutat. Res. 584, 1–256. 10.1016/j.mrgentox.2005.02.004 15979392

[B111] Kohl Y. Rundén-Pran E. Mariussen E. Hesler M. El Yamani N. Longhin E. M. (2020). Genotoxicity of nanomaterials: advanced *in vitro* models and high throughput methods for human hazard assessment—a review. Nanomaterials 10, 1911. 10.3390/nano10101911 32992722 PMC7601632

[B112] Kour J. Ali M. N. Ganaie H. A. Tabassum N. (2017). Amelioration of the cyclophosphamide induced genotoxic damage in mice by the ethanolic extract of *Equisetum arvense* . Toxicol. Rep. 4, 226–233. 10.1016/j.toxrep.2017.05.001 28959643 PMC5615123

[B204] Kremer D. Kosir I. J. Kosalec I. Koncic M. Z. Potocnik T. Cerenak A. (2013). Investigation of chemical compounds, antioxidant and antimicrobial properties of teucrium arduini L. (lamiaceae). Curr. Drug Targets 14 (9), 1006–1014. 10.2174/1389450111314090009 23597042

[B113] Kuhn A. W. Tedesco M. Laughinghouse H. D. Flores F. C. Silva C. D. Canto-Dorow T. S. (2015). Mutagenic and antimutagenic effects of Eugenia uniflora L. by the Allium cepa L. test. Caryologia 68, 25–30. 10.1080/00087114.2014.998525

[B208] Kumar G. Jayaveera K. Ashok C. K. Bharathi T. Umachigi S. P. Vrushabendra S. (2008). Evaluation of antioxidant and antiacne properties of terpenoidal fraction of *Hemidesmus indicus* (Indian sarsaparilla). Internet J. Aesthetic and Antiaging Med. 1. 10.5580/1c88

[B114] Kurt A. H. Olutas E. B. Avcioglu F. Karakuş H. Sungur M. A. Kara Oztabag C. (2023). Quercetin- and caffeic acid-functionalized chitosan-capped colloidal silver nanoparticles: one-pot synthesis, characterization, and anticancer and antibacterial activities. Beilstein J. Nanotechnol. 14, 362–376. 10.3762/bjnano.14.31 36998241 PMC10043739

[B115] Lázaro D. C. López Y. I. Vázquez A. I. F. Odio A. D. González J. E. Sánchez L. M. (2010). Genotoxic assessment of aqueous extract of Rhizophora mangle L. (mangle rojo) by spermatozoa head assay. Rev. Cubana Plantas Med. 15, 18–26.

[B116] Lei F. Zhang X. N. Wang W. Xing D. M. Xie W. D. Su H. (2007). Evidence of anti-obesity effects of the pomegranate leaf extract in high-fat diet induced obese mice. Int. J. Obes. 31, 1023–1029. 10.1038/sj.ijo.0803502 17299386

[B117] Li Y. Wang C. L. Wang Y. J. Wang F. F. Guo S. X. Yang J. S. (2009). Four new bibenzyl derivatives from *Dendrobium candidum* . Chem. Pharm. Bull. 57, 997–999. 10.1248/cpb.57.997 19721264

[B118] Lin C. H. Huang C. C. Wang T. W. Wang Y. J. Lin P. H. (2007). Disparity in the induction of glutathione depletion, ROS formation, poly (ADP-ribose) polymerase-1 activation, and apoptosis by quinonoid derivatives of naphthalene in human cultured cells. Chem. Biol. Interact. 165, 200–210. 10.1016/j.cbi.2006.12.005 17224139

[B119] Lin F. Hasegawa M. Kodama O. (2003). Purification and identification of antimicrobial sesquiterpene lactones from yacon (*Smallanthus sonchifolius*) leaves. Biosci. Biotechnol. Biochem. 67, 2154–2159. 10.1271/bbb.67.2154 14586103

[B120] Liu W. Di Giorgio C. Lamidi M. Elias R. Ollivier E. De Méo M. P. (2011). Genotoxic and clastogenic activity of saponins extracted from Nauclea bark as assessed by the micronucleus and the comet assays in Chinese Hamster Ovary cells. J. Ethnopharmacol. 137, 176–183. 10.1016/j.jep.2011.05.005 21600276

[B121] Liu X. Cui C. Zhao M. Wang J. Luo W. Yang B. (2008). Identification of phenolics in the fruit of emblica (Phyllanthus emblica L.) and their antioxidant activities. Food Chem. 109 (4), 909–915. 10.1016/j.foodchem.2008.01.071 26050007

[B122] Malheiros L. C. S. de Mello J. C. P. Barbosa W. L. R. (2015). Phytochemicals – isolation, characterization and role in human health. in Eleutherine plicata – quinones and antioxidant activity (IntechOpen), 323–338.

[B123] Miadokova E. Nadova S. Vlckova V. Duhova V. Kopaskova M. Cipak L. (2008). Antigenotoxic effect of extract from Cynara cardunculus L. Phytother. Res. 22, 77–81. 10.1002/ptr.2268 17724772

[B124] Mićović T. Topalović D. Živković L. Spremo-Potparević B. Jakovljević V. Matić S. (2021). Antioxidant, antigenotoxic and cytotoxic activity of essential oils and methanol extracts of Hyssopus officinalis L. Subsp. aristatus (godr.) nyman (Lamiaceae). Plants 10, 711. 10.3390/plants10040711 33916934 PMC8067569

[B125] Miyata S. Wang L. Y. Yoshida C. Kitanaka S. (2006). Inhibition of cellular proliferation by diterpenes, topoisomerase II inhibitor. Bioorg Med. Chem. 14, 2048–2051. 10.1016/j.bmc.2005.10.059 16314107

[B126] Miyazaki H. Matsuura H. Yanagiya C. Mizutani J. Tsuji M. Ishihara C. (2003). Inhibitory effects of hyssop (Hyssopus officinalis) extracts on intestinal alpha-glucosidase activity and postprandial hyperglycemia. J. Nutr. Sci. Vitaminol. 49, 346–349. 10.3177/jnsv.49.346 14703310

[B127] Moreira Szokalo R. A. Redko F. Ulloa J. Flor S. Tulino M. S. Muschietti L. (2020). Toxicogenetic evaluation of Smallanthus sonchifolius (yacon) as a herbal medicine. J. Ethnopharmacolo 257, 112854. 10.1016/j.jep.2020.112854 32325177

[B128] Moretti M. Cossignani L. Messina F. Dominici L. Villarini M. Curini M. (2013). Antigenotoxic effect, composition and antioxidant activity of Dendrobium speciosum. Food Chem. 140, 660–665. 10.1016/j.foodchem.2012.10.022 23692750

[B129] Muhammad H. Gomes-Carneiro M. R. Poça K. S. De-Oliveira A. C. Afzan A. Sulaiman S. A. (2011). Evaluation of the genotoxicity of Orthosiphon stamineus aqueous extract. J. Ethnopharmacol. 133, 647–653. 10.1016/j.jep.2010.10.055 21044879

[B201] Munari C. C. Alves J. M. Bastos J. K. Tavares D. C. (2010). Evaluation of the genotoxic and antigenotoxic potential of *Baccharis dracunculifolia* extract on V79 cells by the comet assay. J. Appl. Toxicol. 30 (1), 22–28. 10.1002/jat.1467 19701884

[B130] Nascimento M. F. D. Costa W. K. Aguiar J. C. R. O. F. Navarro D. M. D. A. F. Silva M. V. D. Paiva P. M. G. (2024). Essential oil from leaves of *Croton blanchetianus Baill* does not present acute oral toxicity, has antigenotoxic action and reduces neurogenic and inflammatory nociception in mice. J. Ethnopharmacol. 318, e116908. 10.1016/j.jep.2023.116908 37460027

[B131] Navolokin N. Lomova M. Bucharskaya A. Godage O. Polukonova N. Shirokov A. (2023). Antitumor effects of microencapsulated *Gratiola officinalis* extract on breast carcinoma and human cervical cancer cells *in vitro* . Materials 16, 1470. 10.3390/ma16041470 36837099 PMC9960207

[B132] Navolokin N. A. Mudrak D. A. Polukonova N. V. Bucharskaya A. B. Tychina S. A. Korchakov N. V. (2017). Effects of *Gratiola officinalis* L. extract containing flavonoids on pathomorphism of inoculated renal cancer in rats. Eks. Klin. Farmakol. 80, 19–23.

[B133] Nesterkina M. Bilokon S. Alieksieieva T. Kravchenko I. Hirsch A. K. H. (2023). Genotoxic and mutational potential of monocyclic terpenoids (carvacrol, carvone and thymol) in *Drosophila melanogaster* . Toxicol. Rep. 10, 327–333. 10.1016/j.toxrep.2023.02.009 36911165 PMC9996437

[B134] Nguyen T. Talbi H. Hilali A. Anthonissen R. Maes A. Verschaeve L. (2019). *In vitro* toxicity, genotoxicity and antigenotoxicity of *Nigella sativa* extracts from different geographic locations. S Afr. J. Bot. Journa 126, 132–141. 10.1016/j.sajb.2019.02.015

[B135] Nicolella H. D. de Oliveira P. F. Munari C. C. Costa G. F. Moreira M. R. Veneziani R. C. (2014). Differential effect of manool a diterpene from *Salvia officinalis*, on genotoxicity induced by methyl methanesulfonate in V79 and HepG2 cells. Food Chem. Toxicol. 72, 8–12. 10.1016/j.fct.2014.06.025 25007786

[B136] Nicolella H. D. Fernandes G. Ozelin S. D. Rinaldi-Neto F. Ribeiro A. B. Furtado R. A. (2021). Manool, a diterpene from Salvia officinalis, exerts preventive effects on chromosomal damage and preneoplastic lesions. Mutagenesis 36, 177–185. 10.1093/mutage/geab001 33512444

[B137] Niloufer S. Lakshmi L. B. (2021). *In vitro* analysis of phytochemical, anti-oxidant capacity of seed ethanolic extracts of *Sapindus saponaria vahl* and anti-bacterial activity on common dental pathogens Res. RJPTT 14, 351–355. 10.5958/0974-360X.2021.00064.0

[B138] Oberprieler C. (2001). Phylogenetic relationships in *Anthemis* L. (Compositae, Anthemideae) based on nrDNA ITS and cpDNA trnL/trnF IGS sequence variation. Taxon 50, 745–762. 10.2307/1223705

[B139] Odonne G. Valadeau C. Alban-Castillo J. Stien D. Sauvain M. Bourdy G. (2013). Medical ethnobotany of the chayahuita of the paranapura basin (Peruvian amazon). J. Ethnopharmacol. 146, 127–153. 10.1016/j.jep.2012.12.014 23266276

[B140] Ogura R. Ikeda N. Yuki K. Morita O. Saigo K. Blackstock C. (2008). Genotoxicity studies on green tea catechin. Food Chem. Toxicol. 46, 2190–2200. 10.1016/j.fct.2008.02.016 18381228

[B141] Oliveira A. C. Endringer D. C. Araújo R. J. Brandão M. G. Coelho M. M. (2003). The starch from Solanum lycocarpum St. Hill. fruit is not a hypoglycemic agent. Braz J. Med. Biol. Res. 36, 525–530. 10.1590/s0100-879x2003000400017 12700833

[B142] Paun G. Litescu S. C. Neagu E. Tache A. Lucian Radu G. (2014). Evaluation of Geranium spp., *Helleborus* spp. and *Hyssopus* spp. polyphenolic extracts inhibitory activity against urease and α-chymotrypsin. J. Enzyme Inhib. Med. Chem. 29, 28–34. 10.3109/14756366.2012.749399 23317419

[B143] Perucatti A. Genualdo V. Pauciullo A. Iorio C. Incarnato D. Rossetti C. (2018). Cytogenetic tests reveal no toxicity in lymphocytes of rabbit (*Oryctolagus cuniculus*, 2n=44) feed in presence of verbascoside and/or lycopene. Food Chem. Toxicol. 114, 311–315. 10.1016/j.fct.2018.02.053 29496527

[B144] Petreanu M. Ferreira E. K. Sagaz A. P. Vendramini-Costa D. B. Ruiz A. L. De Carvalho J. E. (2015). Uncommon trimethoxylated flavonol obtained from *Rubus rosaefolius* leaves and its antiproliferative activity. Evid. Based Complement. Altern. Med. 2015, 341216. 10.1155/2015/341216 PMC4691624 26788108

[B145] Ping K. Y. Shohaimi S. Sasidharan S. Yusuf U. K. (2017). Genotoxicity of selected Chinese medicinal plants, elephantopus scaber, Glycyrrhiza uralensis and salvia miltiorrhiza on Allium cepa assay. Ann. Pharmacol. Pharm. 2, 1070.

[B146] Prananda A. T. Dalimunthe A. Harahap U. Simanjuntak Y. Peronika E. Karosekali N. E. (2023). Phyllanthus emblica: a comprehensive review of its phytochemical composition and pharmacological properties. Front. Pharmacol. 14, 1288618. 10.3389/fphar.2023.1288618 37954853 PMC10637531

[B147] Prasad A. (2012). Pharmacognostical phytochemical and pharmacological review on bryophyllum pinnata. IJPBA 3, 423–433.

[B149] Quadros Gomes A. R. da Rocha Galucio N. C. de Albuquerque K. C. O. Brígido H. P. C. Varela E. L. P. Castro A. L. G. (2021). Toxicity evaluation of *Eleutherine plicata* Herb. extracts and possible cell death mechanism. Toxicol. Rep. 8, 1480–1487. 10.1016/j.toxrep.2021.07.015 34401358 PMC8353407

[B150] Ramalho C. E. L. Reis D. D. S. Caixeta G. A. B. Oliveira M. C. Silva D. M. F. D. Cruvinel W. M. (2023). Genotoxicity and maternal-fetal safety of the dried extract of leaves of Azadirachta indica A. Juss (Meliaceae) in Wistar rats. J. Ethnopharmacol. 310, 116403. 10.1016/j.jep.2023.116403 36963474

[B151] Rashed K. N. Ćirić A. Glamočlija J. Calhelha R. C. Ferreira I. C. Soković M. (2013). Antimicrobial activity, growth inhibition of human tumour cell lines, and phytochemical characterization of the hydromethanolic extract obtained from Sapindus saponaria L. aerial parts. Biomed. Res. Int. 2013, 659183. 10.1155/2013/659183 24455713 PMC3888673

[B152] Regner G. G. Gianesini J. Von Borowski R. G. Silveira F. Semedo J. G. Ferraz A. deB. (2011). Toxicological evaluation of *Pterocaulon polystachyum* extract: a medicinal plant with antifungal activity. Biomed. Res. Int. 31, 242–249. 10.1016/j.etap.2010.11.003 21787691

[B153] Rodrigues O. G. Falcao B. R. M. Barbosa B. C. (2019). *In vitro* biological activity of the Croton blanchetianus (Baill) essential oil against Rhipicephalus (Boophilus) microplus (Acari: ixodidae). JABB 7, 55–58. 10.7324/JABB.2019.70210

[B154] Saini R. Sharma N. Oladeji O. S. Sourirajan A. Dev K. Zengin G. (2022). Traditional uses, bioactive composition, pharmacology, and toxicology of Phyllanthus emblica fruits: a comprehensive review. J. Ethnopharmacol. 282, 114570. 10.1016/j.jep.2021.114570 34480995

[B155] Santoro A. Bianco G. Picerno P. Aquino R. P. Autore G. Marzocco S. (2008). Verminoside- and verbascoside-induced genotoxicity on human lymphocytes: involvement of PARP-1 and p53 proteins. Toxicol. Lett. 178, 71–76. 10.1016/j.toxlet.2008.02.006 18395372

[B156] Santos F. J. Moura D. J. Péres V. F. Sperotto A. R. Caramão E. B. Cavalcante A. A. (2012). Genotoxic and mutagenic properties of Bauhinia platypetala extract, a traditional Brazilian medicinal plant. J. Ethnopharmacol. 144, 474–482. 10.1016/j.jep.2012.08.047 23041699

[B157] Santos K. S. Barbosa A. M. Freitas V. Muniz A. V. C. S. Mendonça M. C. Calhelha R. C. (2018). Antiproliferative activity of neem leaf extracts obtained by a sequential pressurized liquid extraction. Pharmaceuticals 11, 76. 10.3390/ph11030076 30061479 PMC6160913

[B158] Sanz-Serrano J. Vettorazzi A. Muruzabal D. López de Cerain A. Azqueta A. (2021). *In vitro* genotoxicity assessment of functional ingredients: DHA, rutin and α-tocopherol. Food Chem. Toxicol. 153, 112237. 10.1016/j.fct.2021.112237 33894296

[B159] Saravanan V. Murugan S. S. Kumaravel T. S. (2020). Genotoxicity studies with an ethanolic extract of Kalanchoe pinnata leaves. MRGTEM 856-857, 503229. 10.1016/j.mrgentox.2020.503229 32928369

[B161] Schorr K. Merfort I. da Costa F. B. (2007). A novel dimeric melampolide and further terpenoids from Smallanthus sonchifolius (Asteraceae) and the inhibition of the transcription factor NF-κB. NPC 2. 10.1177/1934578X0700200404

[B162] Shetty T. K. Satav J. G. Nair C. K. (2005). Radiation protection of DNA and membrane *in vitro* by extract of *Hemidesmus indicus* . Phytother. Res. 19, 387–390. 10.1002/ptr.1470 16106384

[B163] Silva S. Gomes L. Leitao F. Coelho A. V. Boas L. V. (2006). Phenolic compounds and antioxidant activity of Olea europaea L. Fruits and leaves. FSTI. 12, 385–395. 10.1177/1082013206070166

[B164] Slapsytė G. Dedonytė V. Adomėnienė A. Lazutka J. R. Kazlauskaitė J. Ragazinskienė O. (2019). Genotoxic properties of Betonica officinalis, *Gratiola officinalis, Vincetoxicum luteum* and *Vincetoxicum hirundinaria* extracts. Food Chem. Toxicol. 134, e110815. 10.1016/j.fct.2019.110815 31520668

[B205] Soares V. C. Varanda E. A. Raddi M. S. (2006). *In vitro* basal and metabolism-mediated cytotoxicity of flavonoids. Food Chem. Toxicol. 44 (6), 835–838. 10.1016/j.fct.2005.11.006 16376008

[B167] Souza A. B. de Souza M. G. Moreira M. A. Moreira M. R. Furtado N. A. Martins C. H. (2011). Antimicrobial evaluation of diterpenes from *Copaifera langsdorffii oleoresin* against periodontal anaerobic bacteria. Molecules 16, 9611–9619. 10.3390/molecules16119611 22101836 PMC6264602

[B168] Speroni E. Cervellati R. Govoni P. Guizzardi S. Renzulli C. Guerra M. C. (2003). Efficacy of different Cynara scolymus preparations on liver complaints. J. Ethnopharmacol. 86, 203–211. 10.1016/s0378-8741(03)00076-x 12738088

[B169] Sponchiado G. Adam M. L. Silva C. D. Soley B. S. de Mello-Sampayo C. Cabrini D. A. (2016). Quantitative genotoxicity assays for analysis of medicinal plants: a systematic review. J. Ethnopharmacol. 178, 289–296. 10.1016/j.jep.2015.10.026 26680588

[B170] Stoczynska K. Powroźnik B. Pękala E. Waszkielewicz A. M. (2014). Antimutagenic compounds and their possible mechanisms of action. J. Appl. Genet. 55, 273–285. 10.1007/s13353-014-0198-9 24615570 PMC3990861

[B171] Sugahara S. Ueda Y. Fukuhara K. Kamamuta Y. Matsuda Y. Murata T. (2015). Antioxidant effects of herbal tea leaves from yacon (*Smallanthus sonchifolius*) on multiple free radical and reducing power assays, especially on different superoxide anion radical generation systems. J. Food Sci. 80, C2420–C2429. 10.1111/1750-3841.13092 26457985

[B172] Sulaiman M. R. Zakaria Z. A. Bujarimin A. S. Somchit M. N. Israf D. A. Moin S. (2008). Evaluation of *Moringa oleifera* aqueous extract for antinociceptive and anti-inflammatory activities in animal models. Pharm. Biol. 46, 838–845. 10.1080/13880200802366710

[B173] Suzanne J. P. L. Berg V. D. Patrizia R. Marelle G. B. Delmulle L. Ivonne M. C. M. R. (2011). Levels of genotoxic and carcinogenic compounds in plant food supplements and associated risk assessment. FNS 2, 989–1010. 10.4236/fns.2011.29134

[B174] Tavares D. C. Munari C. C. Araújo M. G. Beltrame M. C. Furtado M. A. Gonçalves C. C. (2011). Antimutagenic potential of *Solanum lycocarpum* against induction of chromosomal aberrations in V79 cells and micronuclei in mice by doxorubicin. Planta Medica 77, 1489–1494. 10.1055/s-0030-1270886 21384316

[B175] Teshome S. Jaleta A. Jemal M. Abamecha A. Gershe S. (2023). Phytochemical screening and evaluation of antimicrobial activity of Pterolobium stellatum root extract. Pharm. Pharmacol. Int. J. 11, 148–153. 10.15406/ppij.2023.11.00413

[B176] The Royal Botanic Gardens and Domain Trust (2013). The royal botanic Gardens and Domain Trust annual report. Available at: https://www.parliament.nsw.gov.au/tp/files/30105/2013%20Annual%20Report%20LR_Part1.pdf (Accessed January 18, 2024).

[B177] Tolentino F. Araújo P. A. Marques E. deS. Petreanu M. Andrade S. F. Niero R. (2015). *In vivo* evaluation of the genetic toxicity of *Rubus niveus Thunb* (Rosaceae) extract and initial screening of its potential chemoprevention against doxorubicin-induced DNA damage. J. Ethnopharmacol. 164, 89–95. 10.1016/j.jep.2015.02.013 25681544

[B178] Topalović D. Dekanski D. Spremo-Potparević B. Pirković A. Borozan S. Bajić V. (2019). Dry olive leaf extract attenuates DNA damage induced by estradiol and diethylstilbestrol in human peripheral blood cells *in vitro* . Mutat. Res. Genet. Toxicol. Environ. Mutagen 845, e402993. 10.1016/j.mrgentox.2018.12.001 31561897

[B179] Topalović D. Ž. Živković L. Čabarkapa A. Djelić N. Bajić V. Dekanski D. (2015). Dry olive leaf extract counteracts L-thyroxine-induced genotoxicity in human peripheral blood leukocytes *in vitro* . Oxid. Med. Cell Longev. 2015, e762192. 10.1155/2015/762192 PMC4350944 25789081

[B180] Toscano S. Farieri E. Ferrante A. Romano D. (2016). Physiological and biochemical responses in two ornamental shrubs to drought stress. Front. Plant Sci. 7, 645. 10.3389/fpls.2016.00645 27242846 PMC4867676

[B181] Traore F. Gasquet M. Laget M. Guiraud H. Di Giorgio C. Azas N. (2000). Toxicity and genotoxicity of antimalarial alkaloid rich extracts derived from Mitragyna inermis O. Kuntze and Nauclea latifolia. Phytother. Res. 14, 608–611. 10.1002/1099-1573(200012)14:8<608::aid-ptr667>3.0.co;2-d 11113997

[B182] Trendafilova A. Todorova M. Genova V. Peter S. Wolfram E. Danova K. (2018). Phenolic profile of Artemisia alba Turra. Chem. Biodivers. 15, e1800109. 10.1002/cbdv.201800109 29772115

[B183] Uddin M. S. Mamun A. A. Hossain M. S. Akter F. Iqbal M. A. Asaduzzaman M. (2016). Exploring the effect of Phyllanthus emblica L. on cognitive performance, brain antioxidant markers and acetylcholinesterase activity in rats: promising natural gift for the mitigation of Alzheimer's disease. Ann. Neurosci. 23 (4), 218–229. 10.1159/000449482 27780989 PMC5075744

[B206] Vaz A. M. S. F. Tozzi A. M. G. A. (2005). Sinopse de Bauhinia sect.Pauletia (Cav.) DC. (Leguminosae-Caesalpinioideae: Cercideae) no Brasil. Revista Brasileira de Botânica 28 (3), 477–491. 10.1590/S0100-84042005000300006

[B185] Velikovi D. Randjelovi N. Risti M. S. Velikovi A. Melcerovi A. A. (2003). Chemical constituents and antimicrobial activity of the ethanol extracts obtained from the flower, leaf and stem of Salvia officinalis L. JSCS 68, 17–24. 10.2298/jsc0301017v

[B186] Venditti A. Bianco A. Frezza C. Conti F. Bini L. Giuliani C. (2015). Essential oil composition, polar compounds, glandular trichomes and biological activity of Hyssopus officinalis subsp. aristatus (Godr.) Nyman from central Italy. Ind. Crops Prod. 77, 353–363. 10.1016/j.indcrop.2015.09.002

[B187] Vergara-Jimenez M. Almatrafi M. M. Fernandez M. L. (2017). Bioactive components in moringa oleifera leaves protect against chronic disease. Antioxidants 6, 91. 10.3390/antiox6040091 29144438 PMC5745501

[B188] Verschaeve L. (2015). Genotoxicity and antigenotoxicity studies of traditional medicinal plants: how informative and accurate are the results? Nat. Prod. Commun. 10, 1489–1493. 10.1177/1934578X1501000843 26434148

[B189] Verschaeve L. Edziri H. Anthonissen R. Boujnah D. Skhiri F. Chehab H. (2017). *In vitro* toxicity and genotoxic activity of aqueous leaf extracts from four varieties of *Olea europea* (L). Pharmacogn. Mag. 13, S63–S68. 10.4103/0973-1296.203980 28479728 PMC5407118

[B190] Verschaeve L. Van Staden J. (2008). Mutagenic and antimutagenic properties of extracts from South African traditional medicinal plants. J. Ethnopharmacol. 119, 575–587. 10.1016/j.jep.2008.06.007 18602977

[B191] Wang D. D. Feng Y. Li Z. Zhang L. Wang S. Zhang C. Y. (2014). *In vitro* and *in vivo* antitumor activity of Bulbus Fritillariae Cirrhosae and preliminary investigation of its mechanism. Nutr. Cancer 66, 441–452. 10.1080/01635581.2013.878737 24579826

[B192] Weissenberg M. (2001). Isolation of solasodine and other steroidal alkaloids and sapogenins by direct hydrolysis-extraction of Solanum plants or glycosides therefrom. Phytochemistry 58, 501–508. 10.1016/s0031-9422(01)00185-6 11557084

[B193] Xiang L. Xing D. Lei F. Wang W. Xu L. Nie L. (2008). Effects of season, variety, and processing method on ellagic acid content in pomegranate leaves. Tsinghua Sci. Technol. 13, 460–465. 10.1016/S1007-0214(08)70074-9

[B194] Xu J. Zhao W. Pan L. Zhang A. Chen Q. Xu K. (2016). Peimine, a main active ingredient of Fritillaria, exhibits anti-inflammatory and pain suppression properties at the cellular level. Fitoterapia 11, 1–6. 10.1016/j.fitote.2016.03.018 27033404

[B195] Yang L. N. Xing J. G. He C. H. Wu T. (2014). The phenolic compounds from Dracocephalum moldavica L. Biochem. Syst. Ecol. 54, 19–22. 10.1016/j.bse.2013.12.009

[B196] Zan M. A. Ferraz A. B. Richter M. F. Picada J. N. de Andrade H. H. Lehmann M. (2013). *In vivo* genotoxicity evaluation of an artichoke (Cynara scolymus L.) aqueous extract. J. Food Sci. 78, T367–T371. 10.1111/1750-3841.12034 23330610

[B197] Zhang Y. Zhao L. Guo X. Li C. Li H. Lou H. (2016). Chemical constituents from Phyllanthus emblica and the cytoprotective effects on H 2 O 2-induced PC12 cell injuries. Arch. Pharmacal Res. 39, 1202–1211. 10.1007/s12272-014-0433-2 24993870

[B199] Zhou J. Ouedraogo M. Qu F. Duez P. (2013). Potential genotoxicity of traditional Chinese medicinal plants and phytochemicals: an overview. Phytother. Res. 27, 1745–1755. 10.1002/ptr.4942 23420770

[B200] Zor M. Aslan E. L. (2020). Assessment of *in vitro* antigenotoxic effect of *Nigella sativa* oil. Turk J. Pharm. Sci1 7, 115–118. 10.4274/tjps.galenos.2020.09471 PMC7227873 32454769

